# Exome-chip meta-analysis identifies novel loci associated with cardiac conduction, including *ADAMTS6*

**DOI:** 10.1186/s13059-018-1457-6

**Published:** 2018-07-17

**Authors:** Bram P. Prins, Timothy J. Mead, Jennifer A. Brody, Gardar Sveinbjornsson, Ioanna Ntalla, Nathan A. Bihlmeyer, Marten van den Berg, Jette Bork-Jensen, Stefania Cappellani, Stefan Van Duijvenboden, Nikolai T. Klena, George C. Gabriel, Xiaoqin Liu, Cagri Gulec, Niels Grarup, Jeffrey Haessler, Leanne M. Hall, Annamaria Iorio, Aaron Isaacs, Ruifang Li-Gao, Honghuang Lin, Ching-Ti Liu, Leo-Pekka Lyytikäinen, Jonathan Marten, Hao Mei, Martina Müller-Nurasyid, Michele Orini, Sandosh Padmanabhan, Farid Radmanesh, Julia Ramirez, Antonietta Robino, Molly Schwartz, Jessica van Setten, Albert V. Smith, Niek Verweij, Helen R. Warren, Stefan Weiss, Alvaro Alonso, David O. Arnar, Michiel L. Bots, Rudolf A. de Boer, Anna F. Dominiczak, Mark Eijgelsheim, Patrick T. Ellinor, Xiuqing Guo, Stephan B. Felix, Tamara B. Harris, Caroline Hayward, Susan R. Heckbert, Paul L. Huang, J. W. Jukema, Mika Kähönen, Jan A. Kors, Pier D. Lambiase, Lenore J. Launer, Man Li, Allan Linneberg, Christopher P. Nelson, Oluf Pedersen, Marco Perez, Annette Peters, Ozren Polasek, Bruce M. Psaty, Olli T. Raitakari, Kenneth M. Rice, Jerome I. Rotter, Moritz F. Sinner, Elsayed Z. Soliman, Tim D. Spector, Konstantin Strauch, Unnur Thorsteinsdottir, Andrew Tinker, Stella Trompet, André Uitterlinden, Ilonca Vaartjes, Peter van der Meer, Uwe Völker, Henry Völzke, Melanie Waldenberger, James G. Wilson, Zhijun Xie, Folkert W. Asselbergs, Marcus Dörr, Cornelia M. van Duijn, Paolo Gasparini, Daniel F. Gudbjartsson, Vilmundur Gudnason, Torben Hansen, Stefan Kääb, Jørgen K. Kanters, Charles Kooperberg, Terho Lehtimäki, Henry J. Lin, Steven A. Lubitz, Dennis O. Mook-Kanamori, Francesco J. Conti, Christopher H. Newton-Cheh, Jonathan Rosand, Igor Rudan, Nilesh J. Samani, Gianfranco Sinagra, Blair H. Smith, Hilma Holm, Bruno H. Stricker, Sheila Ulivi, Nona Sotoodehnia, Suneel S. Apte, Pim van der Harst, Kari Stefansson, Patricia B. Munroe, Dan E. Arking, Cecilia W. Lo, Yalda Jamshidi

**Affiliations:** 1 Genetics Research Centre, Molecular and Clinical Sciences Institute, St George’s University of London, London, SW17 0RE UK; 2 Department of Public Health and Primary Care, MRC/BHF Cardiovascular Epidemiology Unit, University of Cambridge, Strangeways Research Laboratory, Worts’ Causeway, Cambridge, CB1 8RN UK; 3 Department of Biomedical Engineering, Cleveland Clinic Lerner Research Institute, Cleveland, OH 44195 USA; 4 Department of Medicine, Cardiovascular Health Research Unit, University of Washington, Seattle, WA 98101 USA; 5 deCODE genetics/Amgen, Inc., 101 Reykjavik, Iceland; 6 Clinical Pharmacology, William Harvey Research Institute, Barts and The London School of Medicine and Dentistry, Queen Mary University of London, London, EC1M 6BQ UK; 7 NIHR Barts Cardiovascular Biomedical Research Centre, Barts and The London School of Medicine and Dentistry, Queen Mary University of London, London, EC1M 6BQ UK; 8 Predoctoral Training Program in Human Genetics, McKusick-Nathans Institute of Genetic Medicine, Johns Hopkins University School of Medicine, Baltimore, MD 21205 USA; 9 Department of Medical Informatics Erasmus MC - University Medical Center, P.O. Box 2040, Rotterdam, 3000 CA The Netherlands; 10 The Novo Nordisk Foundation Center for Basic Metabolic Research, Faculty of Health and Medical Sciences, University of Copenhagen, 2100 Copenhagen, Denmark; 11 Institute for Maternal and Child Health - IRCCS “Burlo Garofolo”, 34137 Trieste, Italy; 12 Institute of Cardiovascular Science, University College London, London, WC1E 6BT UK; 13 Department of Developmental Biology, University of Pittsburgh School of Medicine, Pittsburgh, PA 15201 USA; 14 Division of Public Health Sciences, Fred Hutchinson Cancer Research Center, Seattle, WA 98109 USA; 15 Department of Cardiovascular Sciences, University of Leicester, Cardiovascular Research Centre, Glenfield Hospital, Leicester, LE3 9QP UK; 16 Leicester NIHR Biomedical Research Centre, Glenfield Hospital, Leicester, LE3 9QP UK; 17 Cardiovascular Department, Ospedali Riuniti and University of Trieste, 34100 Trieste, Italy; 18 CARIM School for Cardiovascular Diseases, Maastricht Center for Systems Biology (MaCSBio), and Department of Biochemistry, Maastricht University, Universiteitssingel 60, Maastricht, 6229 ER The Netherlands; 19 Department of Epidemiology, Genetic Epidemiology Unit, Erasmus University Medical Center, Rotterdam, The Netherlands; 20 Department of Clinical Epidemiology, Leiden University Medical Center, Leiden, 2300RC The Netherlands; 21 Department of Medicine, Section of Computational Biomedicine, Boston University School of Medicine, Boston, MA 02118 USA; 22 Biostatistics Department, Boston University School of Public Health, Boston, MA 02118 USA; 23 Department of Clinical Chemistry, Fimlab Laboratories, 33520 Tampere, Finland; 24 Department of Clinical Chemistry, Faculty of Medicine and Life Sciences, University of Tampere, 33014 Tampere, Finland; 25 Medical Research Council Human Genetics Unit, Institute of Genetics and Molecular Medicine, University of Edinburgh, Edinburgh, EH4 2XU UK; 26 Department of Data Science, School of Population Health, University of Mississippi Medical Center, Jackson, MS 39216 USA; 27 Institute of Genetic Epidemiology, Helmholtz Zentrum München - German Research Center for Environmental Health, Neuherberg, Germany; 28 Department of Medicine I, University Hospital Munich, Ludwig-Maximilians University, Munich, Germany; 29 German Centre for Cardiovascular Research (DZHK); partner site: Munich Heart Alliance, Munich, Germany; 30 Mechanical Engineering Department, University College London, London, WC1E 6BT UK; 31 Barts Heart Centre, St Bartholomews Hospital, London, EC1A 7BE UK; 32 Institute of Cardiovascular and Medical Sciences, University of Glasgow, BHF GCRC, Glasgow, G12 8TA UK; 33 Center for Human Genetic Research, Massachusetts General Hospital, Boston, MA 02114 USA; 34 Program in Medical and Population Genetics, Broad Institute of Harvard and MIT, Cambridge, MA 02142 USA; 35 Division Heart & Lungs, Department of Cardiology, University Medical Center Utrecht, University of Utrecht, Utrecht, the Netherlands; 36 Icelandic Heart Association, 201 Kopavogur, Iceland; 37 Department of Cardiology, Faculty of Medicine, University of Iceland, 101 Reykjavik, Iceland; 38 University Medical Center Groningen, University of Groningen, Groningen, The Netherlands; 39 Cardiovascular Research Center and Center for Human Genetic Research, Massachusetts General Hospital, Boston, MA 2114.0 USA; 40 Interfaculty Institute for Genetics and Functional Genomics, University Medicine and Ernst-Moritz-Arndt-University Greifswald, 17475 Greifswald, Germany; 41 DZHK (German Centre for Cardiovascular Research); Partner site Greifswald, 17475 Greifswald, Germany; 42 Department of Epidemiology, Rollins School of Public Health, Emory University, Atlanta, GA 30322 USA; 43 Department of Medicine, Landspitali University Hospital, 101 Reykjavik, Iceland; 44 Julius Center for Health Sciences and Primary Care, University Medical Center Utrecht, Utrecht, Netherlands; 45 Institute of Cardiovascular and Medical Sciences, College of Medical, Veterinary and Life Sciences, University of Glasgow, Glasgow, UK; 46 Erasmus MC - University Medical Center Rotterdam, P.O. Box 2040, Rotterdam, 3000 CA The Netherlands; 47 Cardiovascular Research Center, Massachusetts General Hospital, Charlestown, MA 02114 USA; 48 The Institute for Translational Genomics and Population Sciences and Department of Pediatrics, Los Angeles Biomedical Research Institute at Harbor-UCLA Medical Center, Torrance, CA USA; 49 Division of Genomic Outcomes, Department of Pediatrics, Harbor-UCLA Medical Center, Torrance, CA 90502 USA; 50 Department of Internal Medicine B - Cardiology, Pneumology, Infectious Diseases, Intensive Care Medicine, University Medicine Greifswald, 17475 Greifswald, Germany; 51 Laboratory of Epidemiology and Population Sciences, National Institute on Aging, Intramural Research Program, National Institutes of Health, Bethesda, MD 20892 USA; 52 Cardiovascular Health Research Unit and the Department of Epidemiology, University of Washington, Seattle, WA 98101 USA; 53 Department of Cardiology, Leiden University Medical Center, Leiden, 2300RC The Netherlands; 54 Durrer Center for Cardiogenetic Research, Amsterdam, The Netherlands; 55 Interuniversity Cardiology Institute of The Netherlands, Utrecht, The Netherlands; 56 Department of Clinical Physiology, Tampere University Hospital, 33521 Tampere, Finland; 57 Department of Clinical Physiology, Faculty of Medicine and Life Sciences, University of Tampere, 33014 Tampere, Finland; 58 Department of Medical Informatics, Erasmus University Medical Center, Rotterdam, The Netherlands; 59 Division of Nephrology & Hypertension, Internal Medicine, School of Medicine, University of Utah, Salt Lake City, UT 84109 USA; 60 Research Centre for Prevention and Health, Capital Region of Denmark, 2600 Glostrup, Denmark; 61 Department of Clinical Experimental Research, Rigshospitalet, 2600 Glostrup, Denmark; 62 Department of Clinical Medicine, Faculty of Health and Medical Sciences, University of Copenhagen, 2200.0 Copenhagen, Denmark; 63 Division of Cardiovascular Medicine, Stanford University, Stanford, CA 94305 USA; 64 Institute of Epidemiology, Helmholtz Zentrum München - German Research Center for Environmental Health, Neuherberg, Germany; 65 German Center for Diabetes Research, Neuherberg, Germany; 66 Faculty of Medicine, University of Split, Split, Croatia; 67 Cardiovascular Health Research Unit, Departments of Medicine, Epidemiology, and Health Services, University of Washington, Seattle, WA 98101 USA; 68 Kaiser Permanente Washington Health Research Institute, Seattle, WA 98101 USA; 69 Department of Clinical Physiology and Nuclear Medicine, Turku University Hospital, 20521 Turku, Finland; 70 Research Centre of Applied and Preventive Cardiovascular Medicine, University of Turku, 20014 Turku, Finland; 71 Department of Biostatistics, University of Washington, Seattle, WA 98195 USA; 72 The Institute for Translational Genomics and Population Sciences and Departments of Pediatrics and Medicine, Los Angeles Biomedical Research Institute at Harbor-UCLA Medical Center, Torrance, CA 90502 USA; 73 Epidemiological Cardiology Research Center (EPICARE), Wake Forest School of Medicine, Winston-Salem, NC 27157 USA; 74 Department of Twin Research and Genetic Epidemiology, King’s College London, London, UK; 75 Chair of Genetic Epidemiology, IBE, Faculty of Medicine, LMU Munich, Munich, Germany; 76 Faculty of Medicine, University of Iceland, 101 Reykjavik, Iceland; 77 Department of Gerontology and Geriatrics, Leiden University Medical Center, Leiden, 2300RC The Netherlands; 78 Human Genotyping Facility Erasmus MC - University Medical Center Rotterdam, P.O. Box 2040, Rotterdam, 3000 CA The Netherlands; 79 Institute for Community Medicine, University Medicine Greifswald, 17475 Greifswald, Germany; 80 Research unit of Molecular Epidemiology, Helmholtz Zentrum München - German Research Center for Environmental Health, Neuherberg, Germany; 81 Department of Physiology and Biophysics, University of Mississippi Medical Center, Jackson, MS 39216 USA; 82 TCM Clinical Basis Institute, Zhejiang Chinese Medical University, Hangzhou, 310000 Zhejiang China; 83 Durrer Center for Cardiovascular Research, Netherlands Heart Institute, Utrecht, the Netherlands; 84 Institute of Cardiovascular Science, Faculty of Population Health Sciences, University College London, London, UK; 85 Farr Institute of Health Informatics Research and Institute of Health Informatics, University College London, London, UK; 86 Department of Medical, Surgical and Health Sciences, University of Trieste, 34100 Trieste, Italy; 87 Division of Experimental Genetics, Sidra Medical and Research Center, Doha, Qatar; 88 School of Engineering and Natural Sciences, University of Iceland, 101 Reykjavik, Iceland; 89 Laboratory of Experimental Cardiology, University of Copenhagen, 2200 Copenhagen, Denmark; 90 Division of Medical Genetics, Department of Pediatrics, Harbor-UCLA Medical Center, Torrance, 90502 USA; 91 Department of Public Health and Primary Care, Leiden University Medical Center, Leiden, 2300RC The Netherlands; 92 Dubowitz Neuromuscular Centre, Institute of Child Health, University College London, 30 Guilford Street, London, WC1N 1EH UK; 93 Center for Human Genetic Research and Cardiovascular Research Center, Harvard Medical School and Massachusetts General Hospital, Boston, MA 02114 USA; 94 Usher Institute of Population Health Sciences and Informatics, University of Edinburgh, Edinburgh, EH8 9AG UK; 95 Division of Population Health Sciences, Ninewells Hospital and Medical School, University of Dundee, Dundee, DD1 9SY UK; 96 Department of Epidemiology Erasmus MC - University Medical Center Rotterdam, P.O. Box 2040, Rotterdam, 3000 CA The Netherlands; 97 Division of Cardiology, Departments of Medicine and Epidemiology, University of Washington, Seattle, WA 98101 USA; 98 Department of Genetics, University of Groningen, University Medical Center Groningen, Groningen, The Netherlands; 99 McKusick-Nathans Institute of Genetic Medicine, Johns Hopkins University School of Medicine, Baltimore, MD 21205 USA; 100 Genetics Research Centre, Molecular and Clinical Sciences Institute, St George’s University of London, London, UK

**Keywords:** Exome chip, Conduction, *ADAMTS6*, Meta-analysis

## Abstract

**Background:**

Genome-wide association studies conducted on QRS duration, an electrocardiographic measurement associated with heart failure and sudden cardiac death, have led to novel biological insights into cardiac function. However, the variants identified fall predominantly in non-coding regions and their underlying mechanisms remain unclear.

**Results:**

Here, we identify putative functional coding variation associated with changes in the QRS interval duration by combining Illumina HumanExome BeadChip genotype data from 77,898 participants of European ancestry and 7695 of African descent in our discovery cohort, followed by replication in 111,874 individuals of European ancestry from the UK Biobank and deCODE cohorts. We identify ten novel loci, seven within coding regions, including *ADAMTS6*, significantly associated with QRS duration in gene-based analyses. *ADAMTS6* encodes a secreted metalloprotease of currently unknown function. In vitro validation analysis shows that the QRS-associated variants lead to impaired *ADAMTS6* secretion and loss-of function analysis in mice demonstrates a previously unappreciated role for *ADAMTS6* in connexin 43 gap junction expression, which is essential for myocardial conduction.

**Conclusions:**

Our approach identifies novel coding and non-coding variants underlying ventricular depolarization and provides a possible mechanism for the *ADAMTS6*-associated conduction changes.

**Electronic supplementary material:**

The online version of this article (10.1186/s13059-018-1457-6) contains supplementary material, which is available to authorized users.

## Background

In the heart, the ventricular conduction system propagates the electrical impulses that coordinate ventricular chamber contraction. The QRS interval on an electrocardiogram (ECG) is used clinically to quantify duration of ventricular depolarization in the heart. Prolonged QRS duration is an independent predictor of mortality in both the general population [1–4] and in patients with cardiac disease [5–10].

QRS interval duration is a quantitative trait influenced by multiple genetic and environmental factors and is known to be influenced by both age and gender [11, 12]. The heritability of QRS duration is estimated to be 35–55% from twin and family studies [13–16].

We previously performed a genome-wide association meta-analysis in 40,407 individuals and identified 22 genetic loci associated with QRS duration [17]. The QRS-associated loci highlighted novel biological processes such as kinase inhibitors, but also pointed to genes with established roles in ventricular conduction such as sodium channels, transcription factors, and calcium-handling proteins. However, the common risk variants identified in genome-wide association studies (GWAS) reside overwhelmingly in regulatory regions, making inference of the underlying causative genes difficult. Furthermore, as with most complex traits, the variants discovered to date explain only a small proportion of the total heritability (the “missing heritability” paradigm), suggesting additional variants are yet to be identified. In fact, the role of rare and low frequency variants, which cannot currently be detected using standard genome-wide single nucleotide polymorphism (SNP) chip arrays, have not been fully investigated. Here we used the Illumina HumanExome BeadChip to focus on rare (MAF < 1%), low frequency (MAF = 1–5%), and common (MAF ≥ 5%) putative functional coding variation associated with changes in ventricular depolarization.

## Results and Discussion

We combined genotype data from 77,898 participants of European ancestry and 7695 of African descent participating in the Cohorts for Heart and Aging Research in Genomic Epidemiology (CHARGE) Exome-Chip EKG consortium (Additional file 1: Table S1). A total of 228,164 polymorphic markers on the exome-chip array passed quality control and were used as a basis for our analyses. Through single variant analysis in the combined European and African datasets, we identified 34 variants across 28 loci associated with QRS duration that passed the exome-chip-wide significance threshold (*P* < 6.17 × 10^−8^ for single variants [Table 1, Additional file 2: Figure S1]). Eight of the identified loci were novel and five of these were driven by low frequency (MAF < 5%) and common (MAF ≥ 5%) non-synonymous coding variation. We confirmed 20 of the 29 previously identified QRS duration loci [14, 17–19], the remaining loci were not covered by the Exome-Chip and/or did not pass quality control (QC) (Additional file 1: Table S2). As might be anticipated when combining two ancestries in association analyses, we detected heterogeneity of effects for one variant (Cochran’s heterogeneity *P* < 1.47 × 10^−3^, a Bonferroni corrected *P* value of α=0.05/34 variants), Additional file 1: Table S2). We did not observe evidence for inflation of test statistics for any of the analyses (λ_GC_ = 1.049, European and African ancestries, combined, Additional file 2: Figure S2, individual ancestry results, Additional file 2: Figures S3–S6). We next sought to replicate the 34 lead variants of our 28 loci in a replication meta-analysis of 111,874 individuals from the UK Biobank [20] and deCODE genetics [21] cohorts. In the replication meta-analysis, 30 lead variants for 25 loci replicated (*P* ≤ 1.47 × 10^−3^ = 0.05/34 variants), seven of which were novel, ten of which are known (Additional file 1: Table S2). The remaining four variants that did not replicate in UK Biobank encompass two previously established loci (one in locus *SCN5A*/*SCN10A* for which the other five variants replicated) and two novel loci (*SENP2*, *IGF1R*). This is likely due to differences in phenotype acquisition methods (UK Biobank having exercise ECGs measured), though effect size directions between discovery and replication remained consistent and *P* values of non-replicating variants were all below nominal significance (*P* < 0.05).

**Table 1:** Lead SNPs for 28 loci identified for QRS duration in a combined European and African American ancestry meta-analysis

Locus	Band	dbSNPID	A1/A2	cMAF	beta(se)	*P*	n	Nearest gene	Annotation
Novel loci									
1	2q31.2	rs17362588	A/G	0.081	0.52 (0.08)	4.20 × 10^−11^	85,593	*CCDC141*	Non-synonymous
2	3p22.2	rs116202356	A/G	0.015	− 1.63 (0.17)	1.23 × 10^−20^	85,593	*DLEC1*	Non-synonymous
3	3q27.2	rs6762208	A/C	0.357	− 0.31 (0.05)	3.45 × 10^−12^	85,593	*SENP2*	Non-synonymous
4	6q22.32	rs4549631	C/T	0.481	0.28 (0.04)	5.56 × 10^−11^	85,593	*PRELID1P1*	Intergenic
5	8q24.13	rs16898691	G/C	0.040	− 0.92 (0.11)	5.71 × 10^−16^	79,976	*KLHL38*	Non-synonymous
6	12q13.3	rs2926743	A/G	0.257	− 0.32 (0.05)	9.40 × 10^−11^	85,593	*NACA*	Non-synonymous
7	15q26.3	rs4966020	G/A	0.387	− 0.27 (0.04)	2.99 × 10^−9^	85,593	*IGF1R*	Intronic
8	20p12.3	rs961253	A/C	0.357	0.30 (0.04)	1.20 × 10^−11^	85,593	*CASC20*	Intergenic
Previously identified loci									
9	1p32.3	rs11588271	A/G	0.333	− 0.34 (0.05)	7.59 × 10^−14^	85,593	*CDKN2C*	Intergenic
10	1p13.1	rs4074536	C/T	0.305	− 0.29 (0.05)	8.27 × 10^−10^	85,593	*CASQ2*	Non-synonymous
11	2p22.2	rs7562790	G/T	0.424	0.37 (0.04)	4.34 × 10^−17^	85,593	*CRIM1*	Intronic
12	2p22.2	rs17020136	C/T	0.185	0.38 (0.07)	1.02 × 10^−8^	59,876	*HEATR5B*	Intronic
13	3p22.2	rs6795970	A/G	0.371	0.80 (0.05)	9.19 × 10^−70^	85,593	*SCN10A*	Non-synonymous
14	3p21.1	rs4687718	A/G	0.164	− 0.36 (0.06)	1.19 × 10^−8^	83,134	*TKT*	Intronic
15	5q33.2	rs13165478	A/G	0.377	− 0.68 (0.04)	6.74 × 10^−52^	85,593	*HAND1*	Intergenic
16	6p21.2	rs9470361	A/G	0.249	0.84 (0.05)	1.21 × 10^−63^	85,593	*CDKN1A*	Intergenic
17	6q22.31	rs11153730	C/T	0.475	0.56 (0.04)	1.99 × 10^−38^	85,593	*SLC35F1*	Intergenic
18	7p14.2	rs1362212	A/G	0.144	0.55 (0.06)	1.22 × 10^−18^	85,593	*TBX20*	Intergenic
19	7p12.3	rs7784776	G/A	0.397	0.27 (0.04)	1.18 × 10^−9^	85,593	*IGFBP3*	Intergenic
20	7q31.2	rs3807989	A/G	0.427	0.40 (0.04)	2.14 × 10^−19^	85,593	*CAV1*	Intronic
21	12q24.21	rs3825214	G/A	0.200	0.46 (0.05)	1.10 × 10^−17^	85,593	*TBX5*	Intronic
22	12q24.21	rs7966651	T/C	0.270	− 0.38 (0.05)	6.74 × 10^−15^	85,593	*TBX3*	Intergenic
23	13q22.1	rs1886512	A/T	0.380	− 0.36 (0.05)	3.17 × 10^−13^	70,887	*KLF12*	Intronic
24	14q24.2	rs11848785	G/A	0.237	− 0.44 (0.05)	5.59 × 10^−18^	85,593	*SIPA1L1*	Intronic
25	17q21.32	rs17608766	C/T	0.127	0.70 (0.07)	9.81 × 10^−27^	85,593	*GOSR2*	UTR3
26	17q24.2	rs9912468	G/C	0.416	0.43 (0.05)	2.34 × 10^−21^	79,976	*PRKCA*	Intronic
27	18q12.3	rs663651	G/A	0.446	− 0.44 (0.05)	6.59 × 10^−18^	61,604	*SETBP1*	Non-synonymous
28	20q11.22	rs3746435	C/G	0.190	− 0.36 (0.06)	2.67 × 10^−10^	79,976	*MYH7B*	Non-synonymous

Top panel: novel loci; bottom panel: previously identified loci

*Locus* index number for each independent locus, *Band* cytogenetic band in which the lead SNP for the locus resides, *dbSNPID* dbSNP rs-number of the lead SNP of the locus, *A1/A2* coded/non-coded alleles, *cMAF* cumulative minor allele frequency, *beta(se)* effect size (standard error) in ms, *P P* value, *n* total number of individuals analyzed for this variant, *Nearest gene* (nearest) gene, *Annotation* variant function (protein coding)

### Sex-specific associations with QRS duration

Sex differences in QRS duration are well established (men have significantly longer QRS durations than women [22, 23]), and might be attributable to differential effects of genetic variation in men and women. Therefore, we performed sex-stratified association analyses (Additional file 1: Table S3, Additional file 2: Figures S7 and S8). We included only those studies that had both male and female participants to mitigate potential bias due to contributions from single-sex cohorts. In total, up to 31,702 men and 39,907 women were included from both European and African ancestry studies. We found suggestive evidence for a sex-specific locus that was not identified in the combined analysis. The non-synonymous variant rs17265513 (p.Asn310Ser) in *ZHX3* (zinc fingers and homeoboxes 3) showed a significant association only in men (P_male_ = 4.89 × 10^−8^, β(SE) = − 0.52(0.09)), whereas no effect was observed for women (P_female_ = 0.86, β(SE) = − 0.01(0.08)); however, there was no significant difference consistent with an interaction with sex (*P* = 2.3 × 10^−5^). Additionally, no further evidence was observed in the replication analyses alone (P_male_ = 7.95 × 10^−4^, β(SE) = − 0.30(0.09), N_males_ = 50,457), (P_female_ = 3.55 × 10^−2^, β(SE) = − 0.17(0.08), N_females_ = 61,417).

### Association of coding and non-coding variants with QRS duration

Among the eight newly identified loci in the sex-combined analysis, five had lead variants that were non-synonymous: *CCDC141* (Coiled-Coil Domain Containing 141); *KLHL38* (Kelch Like Family Member 38); *DLEC1* (Deleted in Lung and Esophageal Cancer 1); *NACA* (Nascent Polypeptide-Associated Complex Alpha subunit); and *SENP2* (SUMO1/Sentrin/SMT3 Specific Protease 2). Suggestive evidence for association of the same non-synonymous variant in *CCDC141* (rs17362588; *P* = 4.75 × 10^−7^) and an intronic variant in *KLHL38* (rs11991744; *P* = 1.25 × 10^−7^) with QRS duration was shown in two earlier GWAS [24, 25]. *DLEC1* has recently been suggested to have a possible role as a tumor suppressor [26], and while specific roles for *KLHL38* and *CCDC141* (a centrosome associated protein) have not yet been elucidated, they show the highest expression in skeletal and/or cardiac tissue, respectively, among the tissues examined in the Genotype-Tissue Expression (GTEx) Portal database (http://www.gtexportal.org) [27]. Two of the novel loci, *NACA* and *SENP2*, have established roles in cardiac development and dysfunction. *NACA* produces the isoform skNAC (skeletal NACA) and acts as a skeletal muscle- and heart-specific transcription factor and is critical for ventricular cardiomyocyte expansion [28]. Cardiac-specific knockdown of skNAC in a *Drosophila* Hand4.2-Gal4 driver cell-line results in severe cardiac defects [19]. Cardiac-specific overexpression of *SENP2*, a SUMO-specific protease, leads to congenital heart defects and cardiac dysfunction [29].

In the sex-stratified analysis, the association with *ZHX3* (Zinc Fingers and Homeoboxes 3) was also driven by an amino acid changing variant. *ZHX3* encodes a transcriptional repressor whose functions are largely unknown. However, the sex-specific association might be explained by hormonal changes that have previously been hypothesized to explain a variety of sex-specific differences observed in ECG measures and conduction disorders [30, 31]. A sex-specific association of *ZHX3* has also been previously shown for total cholesterol levels (the effect is only significant in men) [32].

We further identified an intronic variant in the *IGF1R* (Insulin Like Growth Factor 1 Receptor) locus and two intergenic variants: rs4549631 at locus 6q22.32 and rs961253 at locus 20p12.3. Interestingly, when queried against results from the GTEx project portal [27] for blood and eight tissues (including adipose [subcutaneous], artery [aorta, coronary, tibial], heart [atrium, appendage, left ventricle], lung, muscle [skeletal], nerve [tibial], skin [sun exposed], and thyroid), the lead intronic variant in *IGF1R* (rs4966020; MAF EA/AA 0.36/0.63) is a left ventricle tissue-specific cis-eQTL (*P* = 2.4 × 10^−7^). The variant is also in strong linkage disequilibrium with the strongest cis-eQTL for this tissue (rs4966021, *P* = 5 × 10^−8^). IGF1R promotes physiological hypertrophy but protects against cardiac fibrosis [33]; the signaling pathways induced by its binding partner, IGF1, regulate contractility, metabolism, hypertrophy, autophagy, senescence, and apoptosis in the heart [34]. The nearest genes for the two intergenic variants are *PRELID1P1* (PRELI Domain Containing 1 Pseudogene 1 [locus 6q22.32]) and *CASC20* (Cancer Susceptibility Candidate 20 [non-protein-coding]; locus 20p12.3)—the former a pseudogene and the latter a non-protein-coding gene, both with currently uncharacterized function.

### Rare *ADAMTS6* variants are associated with QRS duration

By collapsing rare variants in genes as functional units and jointly testing these for association, substantial statistical power-gains can be achieved [35]. We, therefore, performed gene-based analyses using both the Sequence Kernel Association Test (SKAT) (Additional file 1: Table S4) and burden test (T1) (Additional file 1: Table S5), because these tests have optimal power under different scenarios. Analyses were restricted to variants with MAF < 1% in a total of 16,085 genes. One gene-based significant association (*P* < 5.18 × 10^−7^) was identified in *ADAMTS6* (A Disintegrin-Like And Metalloproteinase with Thrombospondin Type 1 Motif 6; *P*_SKAT_ = 8.18 × 10^−8^, Table 2), when including only variants classified as damaging (see “Methods”). Four additional genes showed suggestive evidence of association (*P* < 1 × 10^−4^) (Table 2).

**Table 2:** Gene-based test association results (for genes with variants classified as damaging)

Gene	N_SNPs_	cMAF	beta(se)_T1-Burden_	P_T1-Burden_	P_SKAT_	Protein function	Cardiac-specific involvement
*ADAMTS6*	12	0.0097	− 0.72 (0.23)	1.48 × 10^**−3**^	8.18 × 10^**−8**^	Zinc-dependent protease	–
*CSRP3*	3	0.0048	1.38 (0.31)	9.65 × 10^**−6**^	9.10 × 10^**−6**^	Regulator of myogenesis	Myocyte cytoarchitecture maintenance
*FHOD3*	17	0.0171	0.00 (0.17)	9.86 × 10^**−1**^	1.82 × 10^**−5**^	Actin filament assembly	Myofibril development and repair
*ISM1*	5	0.0037	1.47 (0.36)	5.05 × 10^**−5**^	5.88 × 10^**−5**^	Angiogenesis inhibitor	–
*TBX5*	8	0.0171	− 0.32 (0.17)	5.21 × 10^**−2**^	7.80 × 10^**−5**^	T-box transcription factor	Cardiac development and cell cycle control

Displayed are the top five genes that have the lowest *P* values in the SKAT test (for genes with damaging variants)

*Gene* gene in which variants were collapsed, *N*_*SNPs*_ number of variants used in the collapsed variant test, *cMAF* cumulative minor allele frequency of variants in the test, *beta(se)*_*T1burden*_ effect size (standard error) in ms, *P*_T1-Burden_ *P* value of T1-burden test, *P*_SKAT_ *P* value of SKAT test, *Protein function* function of the protein encoded by respective gene, *Cardiac-specific involvement*, literature support for physiological involvement of the protein in the heart

The *ADAMTS6* gene-based signal is driven by two rare non-synonymous variants: rs61736454 (p.Ser90Leu) and rs114007286 (p.Arg603Trp), which have allele frequencies of 0.0018 and 0.0021, respectively (Additional file 1: Table S6). Notably, a look-up in the independent deCODE QRS duration analysis showed that rs61736454 was highly significant, however not exome-wide ([*P* = 2.65 × 10^−7^, β(SE) = 3.01(0.58)], MAF = 0.002, *N* = 59,903), and was extremely well imputed (info score = 0.995). Importantly, after meta-analysis with discovery exome summary statistics, the signal reached exome-wide significance ([*P* = 8.96 × 10^−13^, β(SE) = 2.75(0.38)], *N* = 145,496), underscoring the robustness of our initial discovery signal driver. Data for rs114007286 were not available. *ADAMTS6* is a highly constrained gene, with a probability of loss of function intolerance score of 1.0 (pLI = 1.0) (Exome Aggregation Consortium [ExAC], Cambridge, MA, USA; http://exac.broadinstitute.org/). The p.Ser90Leu variant lies within the ADAMTS6 propeptide, which is predicted to be important for initiation of folding, because the homologous ADAMTS9 propeptide is an intramolecular chaperone essential for its secretion [36]. The second variant, p.Arg603Trp, is located in the N-terminal-most TSR domain (TSR1) of ADAMTS6. This domain is the target of protein-*O*-fucosylation, which is a QC signal that prevents secretion of ADAMTS proteins that are improperly folded [37].

### ADAMTS6 is necessary for cardiac development and expression of gap junction protein Cx43

**Fig. 1:**
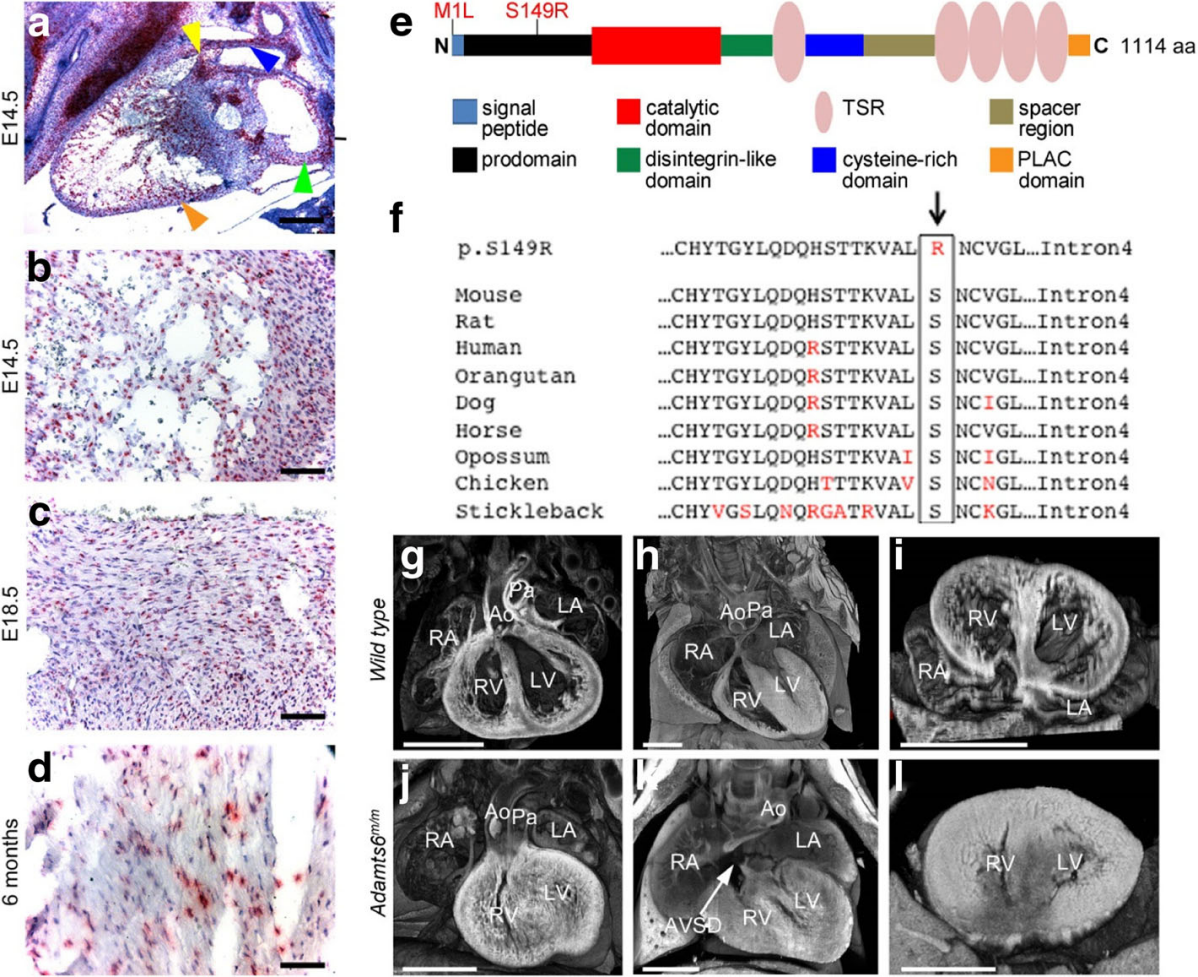
*Adamts6* cardiac expression, sequence conservation, and cardiac anomalies in *Adamts6*-deficient mice. **a**–**d** *Adamts6* (*red punctate signal*) is expressed in the outflow tract (**a**, *blue arrowhead*), heart valves (**a**, *yellow arrowhead*), atria (**a**, *green arrowhead*), and ventricular myocardium (**a**, *orange arrowhead*, **b**-**d**). **e**, **f** *Diagram* of the two *Adamts6* mutant alleles recovered: Met1Ile and Ser149Arg. The sequence alignment shows conservation of the Ser149 residue in ADAMTS6 across species. **g**–**l** Congenital heart defects observed in *Adamts6* Ser149Arg (*Adamts6*^*m/m*^) mutant embryos. A WT mouse heart with normal atrial, ventricular, and outflow tract anatomy (**g**), an intact atrioventricular septum (**d**), and normal ventricular myocardium (**i**). Homozygous *Adamts6* Ser149Arg mutants (*Adamts6*^*m/m*^) exhibit a spectrum of congenital heart defects, such as a double outlet right ventricle (**j**, in which the aorta and pulmonary artery both arise from the right ventricle; see Additional file 3: Video S1) or an atrioventricular septal defect (AVSD) (**k**, in which the atrial and ventricular septa fail to form). Thickening of the ventricular wall is commonly observed, indicating ventricular hypertrophy (**l**). These mutant hearts (**j**–**l**) are shown at embryonic day (E)16.5 but their development is delayed, giving an appearance similar to WT hearts at E14.5 (as shown in (**g**–**i**)). Ao aorta, AVSD atrioventricular septal defect, LA left atrium, LV left ventricle, Pa pulmonary artery, RA right atrium, RV right ventricle. Scale bar: (**a**) 500 μm; (**b**–**d**) 50 μm; (**g**–**l**) 1 mm

ADAMTS6 belongs to a family of metalloproteases that mediates extracellular proteolytic processing of extracellular matrix (ECM) components and other secreted molecules. ADAMTS6 is closely related to ADAMTS10, which interacts with and accelerates assembly of fibrillin-1, mutations in which cause Marfan syndrome [38]. This suggests that ADAMTS6 could regulate cardiac ECM. While no specific ADAMTS6 substrates have been unequivocally identified, it was reported to regulate focal adhesions, epithelial cell–cell interactions, and microfibril assembly in cultured cells [39]. We show by RNA in situ hybridization that *Adamts6* is expressed in the atrioventricular and septal cushions and myocardium of the embryonic heart, with expression persisting into adult ventricular, trabecular, and septal myocardium (Fig. 1a–d).

Mice with recessive *Adamts6* mutations were recovered in a forward genetic screen [40] (Fig. 1e and f). One mutation (p.Met1Ile) affects the start codon and is predicted null. The second mutation (p.Ser149Arg) lies in the propeptide. Both mutations cause prenatal/neonatal lethality with identical congenital heart defect phenotypes (Additional file 1: Table S7), comprising double outlet right ventricle (Fig. 1j, Additional file 3: Video S1), atrioventricular septal defect (Fig. 1k), and ventricular hypertrophy (Fig. 1j and l).

**Fig. 2:**
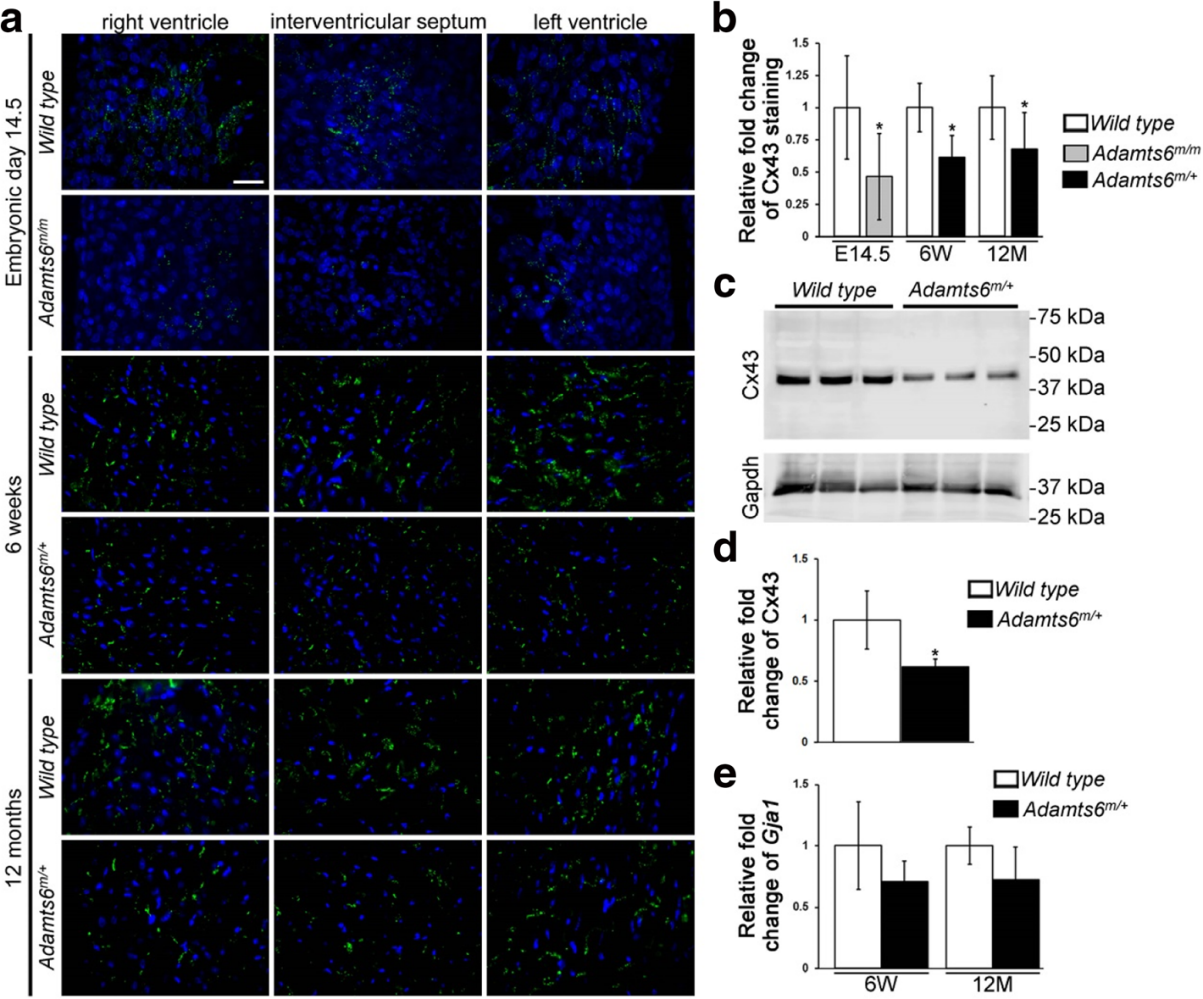
Reduction of Cx43 intercalated disk gap junction staining in *Adamts6*-deficient mice. **a**, **b** Cx43 staining (*green*) (**a**) is reduced throughout ventricular myocardium in embryonic day (E) 14.5 *Adamts6*^*m/m*^ embryos and 6-week and 12-month *Adamts6*^*m/+*^ mice and quantified in (**b**). DAPI (*blue*) was used to visualize cell nuclei. **c**, **d** Representative western blot (**c**) and quantification (**d**) shows reduced Cx43 in three pairs of 6-week *Adamts6*^*m/+*^ and WT myocardium controls. Gapdh was used as a loading control. **e** No change in *Gja1* RNA level in 6-week and 12-month *Adamts6*^*m/+*^ myocardium as compared to control. Scale bar: 50 μm. *P ≤ 0.01. E embryonic, W weeks, M months

Ventricular conduction relies on cardiomyocyte coupling through gap junctions, with connexin 43 (Cx43) being the predominant myocardial gap junction protein in the human and mouse myocardium. *Gja1* (encoding Cx43) knockout mice exhibit slow conduction, QRS prolongation, and increased susceptibility to ventricular arrhythmias [41–43], consistent with its role in mediating electrical coupling required for efficient propagation of ventricular depolarization. While *Adamts6* heterozygous (*Adamts6*^*m/+*^) adult mice are viable and without structural heart defects (Additional file 2: Figure S9), their ventricular myocardium shows reduced Cx43 staining (Fig. 2a and b). Western blot shows reduction of Cx43 protein in the adult *Adamts6*^*m/+*^ myocardium (Fig. 2c and d). Interestingly, parallel quantitative real-time polymerase chain reaction (qRT-PCR) shows unchanged *Gja1* messenger RNA (mRNA) expression (Fig. 2e), suggesting post-transcriptional regulation. Analysis of embryonic day 14.5 homozygote *Adamts6*^*m/m*^ mutants shows Cx43 is completely absent in the ventricular myocardium (Fig. 2a and b). Thus, whereas *Adamts6*^*m/m*^ mice have severe structural heart defects and Cx43 deficiency, *Adamts6*^*m/+*^ hemizygosity leads to reduction in Cx43 expression in the ventricles without defects in cardiac morphogenesis. Together these findings suggest the QRS prolongation in individuals with rare pathogenic *ADAMTS6* variants could arise from impaired myocardial connectivity due to Cx43 reduction.

### Rare *ADAMTS6* coding variants lead to impaired ADAMTS6 secretion

**Fig. 3:**
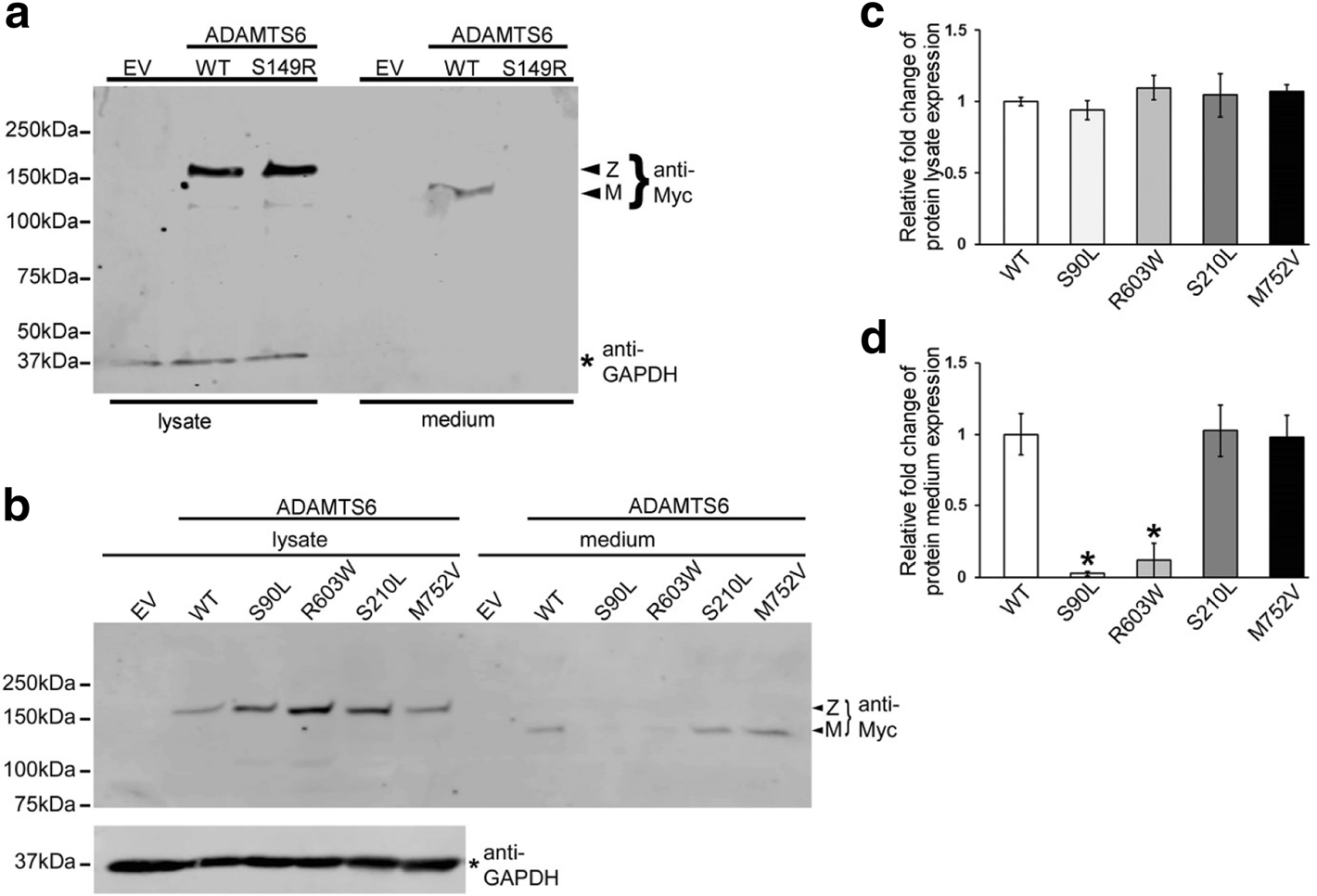
A mouse *Adamts6* ENU mutant and predicted damaging *ADAMTS6* variants have impaired secretion. **a**, **b** Representative western blots using anti-Myc antibody show a major molecular species of 150 kDa in HEK293F cell lysates, corresponding to the ADAMTS6 zymogen (Z). In contrast, the culture medium of cells transfected with WT ADAMTS6 shows a 130 kDa species, corresponding to mature (M, i.e. furin-processed) ADAMTS6. **a** The p.Ser149Arg murine variant is not secreted into the culture medium. **b** The predicted damaging human variants, p.Ser90Leu and p.Arg603Trp, have reduced secretion, whereas the predicted benign variants, p.Ser210Leu and p.Met752Val, are secreted normally. Lysate and medium of HEK293F cells transfected with an empty vector (EV) lack immunoreactivity. The membrane was subsequently re-blotted using an anti-GAPDH monoclonal antibody to demonstrate comparable sample loading. **c**, **d** Densitometry of ADAMTS6 signal in lysates (**c**) and medium (**d**) shows reduced secretion of p.Ser90Leu and p.Arg603Trp variants and normal secretion of p.Ser210Leu and p.Met752Val into the medium, relative to the WT control (*P ≤ 0.01 for n = 3 transfections of each vector)

To determine the functional consequences of the two predicted pathogenic human *ADAMTS6* coding variants from the exome-chip analysis (p.Ser90Leu and p.Arg603Trp), myc-tagged *ADAMTS6* constructs with the variants introduced by site-directed mutagenesis were expressed in HEK293F cells. Western blotting was used to compare the levels of mutant and wild type (WT) myc-tagged ADAMTS6 in the transfected cell lysates and medium. As positive and negative controls, respectively, we transfected the known pathogenic murine variant (p.Ser149Arg) and two rare non-synonymous human *ADAMTS6* variants predicted to be benign (p.Ser210Leu and p.Met752Val). Western blotting confirmed that the *Adamts6* p.Ser149Arg variant was not secreted (Fig. 3a). The predicted human pathogenic variants show much reduced secretion compared to the WT and benign variants (Fig. 3b–d). Significantly, the molecular masses of the secreted p.Ser90Leu and p.Arg603Trp variants observed in cell lysate are comparable to that of the WT protein, indicating normal glycosylation and propeptide excision, which are essential for ADAMTS zymogen conversion to their mature forms [44]. These results suggest that heterozygous individuals have a reduction of secreted ADAMTS6 to 50% of normal, implying reduced proteolytic activity. The resulting disruption of proteolytic remodeling could potentially affect cell–cell and cell–matrix interactions essential for efficient Cx43 gap junction assembly. However, the rs61736454 (p.Ser90Leu) and rs114007286 (p.Arg603Trp) variants were associated with longer and shorter QRS duration, respectively. The reduced secretion observed was more profound for the rs61736454 variant compared to rs114007286, and the assay does not predict what impact a small amount of secreted protein may have, nor how it interacts in the presence of other modifier genes/variants carried by the same individual. Additionally, the two variants might affect overall protein function and interaction with binding partners in different ways.

## Conclusions

In a meta-analysis of data from 77,898 participants of European ancestry and 7695 of African descent in our discovery cohort participating in the Cohorts for Heart and Aging Research in Genomic Epidemiology (CHARGE) Exome-Chip ECG consortium, we identified 28 loci associated with QRS duration. With the addition of 111,874 individuals of European ancestry from the UK Biobank and deCODE cohorts, all 34 variants across the 28 loci passed the exome-chip-wide significance threshold, indicating our results are robust. Furthermore, effect size directions between discovery and replication remained consistent and *P* values of non-replicating variants in the replication analysis alone were all below nominal significance (*P* < 0.05). Novel loci include genes involved in cardiac development and dysfunction, some of which are highly expressed in skeletal and/or cardiac tissue. To establish further evidence for these novel loci and mechanisms underlying each association, future functional experiments are essential.

The present study also highlights the efficacy of large-scale population-based exome-chip analysis for discovery of non-synonymous coding variants with significant functional effects. In gene-based tests, we identified an association between ventricular depolarization and rare non-synonymous variants in *ADAMTS6*, a gene not previously implicated in cardiac conduction. We chose to focus on this novel locus and seek functional validation as the association was driven by multiple rare coding variants that were predicted to be damaging by in silico tools. The coding variants driving the association in the population study and the mutations identified in the mouse forward genetic screen all impair ADAMTS6 secretion, indicating reduction/loss of function. Significantly, although heterozygosity of the variants in mice is not associated with structural heart defects, we detected reduction of Cx43 gap junctions in the ventricular myocardium. Homozygous *Adamts6* mutants show complete loss of Cx43 gap junctions as well as structural heart defects, implying a dosage effect. Together, these findings indicate that ADAMTS6 has a novel role in regulating gap junction-mediated ventricular depolarization, with quantitative reduction in ADAMTS6 causing cardiac conduction perturbation. While our study focuses on cardiac conduction, the findings support the potential broad utility of large-scale exome-chip analysis for interrogating coding variants associated with other physiological or clinical parameters.

## Methods

### Discovery association analyses

#### Study cohorts

All participating studies formed the CHARGE EKG exome-chip consortium, including those belonging to the CHARGE consortium and external studies to investigate the role of functional variation in electrocardiographic traits. Twenty-two cohorts participated in the QRS duration analysis effort representing a maximum total sample size of 85,593 samples, consisting of 77,898 participants of European ancestry (91%) and 7695 of African descent. Individual study details and characteristics are summarized in Additional file 1: Table S1.

#### Phenotype measurements

We analyzed QRS duration measured in milliseconds. In each study, individuals were excluded from the analyses if these had a QRS duration of > 120 ms, atrial fibrillation (AF) on baseline electrocardiogram, a history of myocardial infarction or heart failure, had Wolff–Parkinson–White syndrome (WPW), a pacemaker, or used Class I and class III blocking medications (those medications with prefix C01B* according to the Anatomical Therapeutic Chemical (ATC) Classification System, http://www.whocc.no/atcddd/) [45]. For cohorts that were disease case-control studies, we included only the control subjects in our analyses irrespective of the nature of the case disease.

#### Genotyping and quality control

Each participating study performed genotyping using the Illumina HumanExome BeadChip / HumanCoreExome platforms. Owing to the difficulty of accurately detecting and assign genotype calls for rare variants (MAF < 1%), an initial core set of CHARGE cohorts, comprising approximately 62,000 samples, assembled intensity data into a single project for a joint improved calling. The quality of the joint calling was assessed through investigating the concordance of genotypes in samples having both exome-chip and exome-sequence data, described extensively elsewhere [46, 47]. Using the curated clustering files from the CHARGE central calling effort, several cohorts within our study re-called their genotypes. The remainder of participating studies used either Gencall [48] or zCall [49], or a combination of both. Full details concerning the genotyping and quality control for each cohort are summarized in Additional file 1: Table S1. Individual studies performed sample-level genotype QC filtering for call rate, removing autosomal heterozygosity outliers, gender mismatches, duplicates as established by identity by descent (IBD) analysis, and removed ethnic outliers as determined by multidimensional scaling. Poorly called variants were typically removed by filtering for Hardy-Weinberg equilibrium test *P* value (pHWE), call rate, and filtering removing poorly clustering variants. Each study aligned their data reference strand to the Illumina forward strand using a central SNP allele reference and annotation file (SNP info file) [46] for the Illumina Exome Chip. Variants were all mapped to GRCh37/hg19. Only variants present within the SNP info file were initially considered for analyses, 247,871 in total. Next, we filtered out 9252 variants that failed QC in the joint calling effort, as well as 6591 variants with inconsistent reference alleles across studies (a total of 11,392 unique SNPs), and considered furthermore only autosomal and chromosome X variants, and only those that were polymorphic in our study, leaving an initial set of 228,164 variants for analysis. For our single variant analyses, we only included variants with MAF > 0.012% (equal to a minor allele count [MAC] of 10), 162,199 in total.

#### Statistical methods

All association analyses were carried out using the R-package seqMeta [50]. Each study ran the “prepScores” function and adjusted their analyses for age, gender, body mass index (BMI), height, principal components, and study-specific covariates when appropriate (details in Additional file 1: Table S1). The output of this function is an R “list” object (“a prepScores object”), stored in an .RData file, where each element corresponds to a gene, and contains the scores and MAFs for variants, as well as a matrix of the covariance between the scores at all pairs of SNPs within a gene. All studies performed both gender combined and separated analyses, in addition to separation by ancestry. Using the prepScores objects from each study, we performed meta-analyses using the “singlesnpMeta()” for single variant meta-analyses, and the “burdenMeta” and “skatMeta()” functions of SeqMeta. Coefficients and standard errors from seqMeta can be interpreted as a “one-step” approximation to the maximum likelihood estimates. Ancestry groups were analyzed both separate and combined at the meta-analysis level.

For single variant meta-analyses, we included all variants with a MAC ≥ 10 in order to have well-calibrated type I error rates [51]. Statistical significance was defined using Bonferroni corrections. For single variants, maximally 162,199 variants were included in five separate analyses after filtering for MAC: European and African ancestry separated and combined (n = 3); and sex-stratified analyses (n = 2), resulting in a Bonferroni corrected *P* value of α=0.05 / 162,199 variants / 5 analyses = 6.17 × 10^−8^.

Suggestive sexually dimorphic associations were identified by performing sex-stratified meta-analyses, totaling 39,907 women and 31,702 men, including only from cohorts that had both male and female samples. Variants were deemed to be suggestive sex-specific when reaching below a *P* value threshold of exome-wide significance (*P* < 6.17 × 10^−8^) in one sex and above nominal significance in the other (*P* > 0.05).

For gene-based tests, also performed using seqMeta using the “prepScores” objects from individual cohorts, we assigned variants to genes by annotating all variants on the Exome Chip using ANNOVAR [52] following RefSeq [53] gene definitions mapped to human genome build 37 (hg19). In the collapsed variant tests, we included only variants with MAF < 1% and included only genes for which two or more variants were present (n = 16,085). We performed both SKAT [54] and T1 burden [55] tests, for three different functional sets of variants limited to the following: (I) all variants; (II) missense, nonsense, splice, and indel variants; (III) “damaging”: the same variants as in group II, except for missense only including those that are predicted to be damaging by at least two out of four functional prediction algorithms (Polyphen2 [56], SIFT [57], Mutation Taster [58], and LRT [59]). For the gene-based tests, we used a Bonferroni corrected *P* value significance threshold of α=0.05 / 16,085 genes / 2 different tests / 3 functional variant classes = 5.18 × 10^−7^.

We define a physically independent locus as the genomic region that contains variants within 250 kb on either side of LD-independent lead SNPs (exome-wide significant variants with r2 < 0.1), where LD calculations were based on European ancestry. Following this definition, in certain cases LD-independent lead variants are present in overlapping regions, complicating the definition and reporting of associated genetic loci and harbored genes. Therefore, we annealed loci if LD-independent exome-wide significant variants were < 250 kb from each other. Where lead SNPs from previous analyses were not contained in these regions, we considered these as novel. LD calculations were performed on the Illumina Exome Chip genotype data from the TwinsUK cohort [60] (n = 1194), using PLINK 1.9 [61].

### Replication association analyses

#### Study cohort: UK biobank (UKB)

UK Biobank (www.ukbiobank.ac.uk) is a prospective study of 500,000 volunteers, comprising relatively even numbers of men and women aged 40–69 years old at recruitment, with extensive baseline, and follow-up clinical, biochemical, genetic, and outcome measures. Approximately 95,000 individuals were recruited for a Cardio test using a stationary bicycle in conjunction with a four-lead electrocardiograph device at the initial assessment (2006–2008) and ~ 20,000 individuals performed the test again (the first repeat assessment: 2011–2013). The Cardio test, thereafter known as the exercise test, started with 15 s of rest (pre-test), followed by 6 min of exercise (cycling) with an increasing workload, and a 1-min recovery period without exercise. To improve accuracy, we calculated an average QRS waveform by aligning all QRS complexes present in a window of 15 s from the resting stage. Ectopic beats and artifacts were removed. Then, we calculated the correlation between each individual QRS complex and the average QRS waveform and removed those with a correlation coefficient < 0.8. Finally, we repeated the calculation of the average QRS waveform by only considering those highly correlated individual QRS complexes. The QRS width was measured from the average QRS waveform as the interval between the onset of the Q wave and the end of the S wave. Genotyping was performed by UKB using the Applied Biosystems UK BiLEVE Axiom Array or the UKB AxiomTM Array. Single Nucleotide Variants (SNVs) were imputed centrally by UKB using a merged UK10K sequencing + 1000 Genomes imputation reference panel (https://www.biorxiv.org/content/early/2017/07/20/166298). Following phenotype and genotype QC, a total of 51,971 unrelated individuals of European ancestry remained for analysis. Thirty-four QRS discovery lead variants selected for replication were extracted from UKB imputed files, all being of high quality (Hardy-Weinberg *P* > 1 × 10^−4^ and an info score > 0.5) using QCTOOL v2 and the association analysis was performed using SNPTEST v2.5.4 assuming an additive genetic model.

#### Study cohort: deCODE

ECGs obtained in Landspitali—The National University Hospital of Iceland, Reykjavik, the largest and only tertiary care hospital in Iceland—have been digitally stored since 1998. For this analysis, we used information on mean QRS duration in milliseconds from 151,667 sinus rhythm ECGs from 59,903 individuals. Individuals with permanent pacemakers or history of myocardial infarction, heart failure, atrial fibrillation, or WPW were excluded, as well as ECGs with QRS duration > 120 ms. ECG measurements were adjusted for sex, year of birth, and age at measurement. Due to limited availability of information, height, BMI, or drugs were not accounted for in the analysis. The genotypes in the deCODE study were derived from whole-genome sequencing of 28,075 Icelanders using Illumina standard TruSeq methodology to a mean depth of 35X (SD 8X) with subsequent imputation into 160,000 chip-typed individuals and their close relatives [21]. Selected replication variants from the meta-analysis for association with QRS duration were tested in accounting for relatedness using a mixed effects model as implemented by BOLT-LMM [62] followed by LD score regression [63].

#### Statistical analysis

We first performed a fixed-effects inverse variance weighted meta-analysis combining the summary statistics data from the UKB and deCODE analyses, followed by a combined analysis of the discovery and replication summary statistics using GWAMA v2.2.2 [64].

### Mouse and cell models

#### Western blot analysis

A plasmid vector for expression of the full-length *Adamts6* open reading frame was generated via PCR using Phusion High-Fidelity DNA Polymerase (catalog no. M0530 L; New England Biolabs) and embryonic mouse heart complementary DNA (cDNA) as the template and inserted into PSecTag2B (V900–20; Life Technologies). *ADAMTS6* variants p.Ser90Leu and p.Arg603Trp were created in the *Adamts6* cDNA using Q5 Site-Directed Mutagenesis Kit (catalog no. E0554S; New England BioLabs). Primer sequences used for cloning and mutagenesis are available upon request. Each plasmid insert was verified by sequencing. Human embryonic kidney (HEK293) cells obtained from ATCC were maintained in medium supplemented with 10% fetal bovine serum and 100 U/mL penicillin and 100 μg/mL streptomycin. The constructs were transfected with Lipofectamine 3000 Transfection Kit (catalog no. L3000; Invitrogen) following manufacturer’s instructions. After 72 h in serum-free medium, cell lysates were collected in lysis buffer (0.1% NP-40, 0.01% sodium dodecyl sulfate, and 0.05% sodium deoxycholate in phosphate buffered saline [PBS], pH 7.4). Extracts were electrophoresed by reducing SDS-PAGE on 10% Tris-Glycine gels. Proteins were electroblotted to Immobilon-FL membranes (catalog no. IPFL00010, EMD Millipore), incubated with primary antibody anti-myc (Hybridoma core facility; 1:1000; Cleveland Clinic), anti-GAPDH (catalog no. MAB374; 1:5000; EMD Millipore), and anti-Cx43 (catalog no. C6219; 1:2000; Sigma-Aldrich), overnight at 4 °C, followed by IRDye secondary antibodies goat anti-mouse or anti-rabbit (926–68,170, 827–08365; 1:10000; LI-COR) for 1 h at room temperature and visualized by Odyssey CLx (LI-COR). Band intensity was measured using ImageJ (NIH, Bethesda, MD, USA).

##### Statistics

All values are expressed as mean ± SEM. A paired two-tailed Student’s t-test was used to assess statistical significance.

#### Recovery and phenotyping of *Adamts6* mutant mice

*Adamts6* mutant mice were recovered from a recessive ethynitrosourea (ENU) mouse mutagenesis screen conducted using non-invasive in utero fetal echocardiography [40]. Mutants detected with congenital heart defects by ultrasound imaging were recovered either as fetuses or at term and further analyzed by necropsy, followed by histopathology for detailed analysis of intracardiac anatomy with three-dimensional reconstructions using episcopic confocal microscopy. From the screen, ten independent *Adamts6* mutant lines were recovered, all exhibiting the identical phenotype. Mouse histology, immunostaining and RT-PCR experiments were approved by the Cleveland Clinic Institutional Animal Care and Use Committee (protocol # 2015–1458, IACUC number: 18052990).

#### Mouse mutation recovery

Mutation recovery was conducted by whole-exome capture using SureSelect Mouse All Exon kit V1, with sequencing carried out using Illumina HiSeq 2000 with minimum 50X average coverage (BGI Americas). Sequence reads were aligned to the C57BL/6 J mouse reference genome (mm9) and analyzed using CLCBio Genomic Workbench and GATK software. All homozygous mutations were genotyped across all mutants recovered in the mutant line and only the *Adamts6* mutation was consistently homozygous across all mutants recovered in the line, the pathogenic identifying it as mutation. Of the ten mutant lines, nine were identified to have the same missense mutation (c.C447G: p.S149R), while one mutant line exhibited loss of the start codon (c.G3A: p.M1I) and was confirmed to be null with no *Adamts6* transcripts detected with transcript analysis. The *Adamts6* missense mutation was subsequently identified as a spontaneous mutation in the C57BL/6 J production colony at the Jackson Laboratory.

#### Histology and immunofluorescence staining and RNA in situ hybridization

Tissues were fixed in 4% paraformaldehyde in PBS at 4 °C overnight followed by paraffin embedding. Sections of 7 μm were used for hematoxylin and eosin staining, picrosirius red staining, and immunofluorescence for Cx43 (catalog no. C6219; 1:800; Sigma-Aldrich) followed by secondary goat anti-rabbit antibody (catalog no. 111–035-144; 1:2000; Jackson Immunoresearch Laboratories Inc.). Antigen retrieval, i.e. immersion of slides in citrate-EDTA buffer (10 mM/L citric acid, 2 mM/L EDTA, 0.05% *v*/v Tween-20, pH 6.2) and microwaving for 1.5 min at 50% power four times in a microwave oven with 30-s intervals intervening was used before immunofluorescence. Immunofluorescence was quantified by the ratio of Cx43 signal to DAPI-positive cell nuclei integrated density (ImageJ; National Institutes of Health, *n* = 3, with three samples of each myocardium). *Adamts6* RNA in situ hybridization was performed using RNAScope (Advanced Cell Diagnostics) following the manufacturer’s protocol. Briefly, 7-μm sections were deparaffinized and hybridized to a mouse *Adamts6* probe set (catalog no. 428301; Advanced Cell Diagnostics) using a HybEZ™ oven (Advanced Cell Diagnostics) and the RNAScope 2.5 HD Detection Reagent Kit (catalog no. 322360; Advanced Cell Diagnostics).

#### Quantitative real-time PCR

Total RNA was isolated using TRIzol (catalog no. 15596018, Invitrogen) and 1 μg of RNA was reverse-transcribed into cDNA with SuperScript III Cells Direct cDNA synthesis system (catalog no. 46–6321, Invitrogen). qPCR was performed with Bullseye EvaGreen qPCR MasterMix (catalog no. BEQPCR-S; MIDSCI) using an Applied Biosystems 7500 instrument. The experiments were performed with three independent samples and confirmed reproducibility. *Gapdh* was used as a control for mRNA quantity. The ∆∆Ct method was used to calculate relative mRNA expression levels of target genes. Primer sequences are as follows*: Gapdh*: 5’ TGGAGAAACCTGCCAAGTATGA 3′ and 5’ CTGTTGAAGTCGCAGGAGACA 3′; *Gja1*: 5’ CCTGCTGAGAACCTACATCATC 3′ and 5’CGCCCTTGAAGAAGACATAGAA 3′.

### Web resources

Databases

**Genotype-Tissue Expression (GTEx) Portal database**: http://www.gtexportal.org

Software

**seqMeta**: http://cran.r-project.org/web/packages/seqMeta/

**EasyStrata**: https://cran.r-project.org/web/packages/EasyStrata/

**PLINK 1.9**: https://www.cog-genomics.org/plink

**SNPTEST v2.5.4**: https://mathgen.stats.ox.ac.uk/genetics_software/snptest/snptest.html

**GWAMA v.2.2.2**: https://www.geenivaramu.ee/en/tools/gwama

## Additional files

Additional file 1: **Table S1.** Cohort characteristics. **Table S2.** Single SNP meta-analyses. **Table S3.** Sex-stratified analyses. **Table S4.** SKAT analyses. **Table S5.** T1-burden analyses. **Table S6.** *ADAMTS6* variant details. **Table S7.** Cardiac phenotype distribution in *Adamts6* mutant mice. (XLSX 475 kb)

Additional file 2: **Figure S1.** Manhattan plot for European and African-American ancestry single variant analysis. **Figure S2.** Quantile-quantile plot for European and African-American ancestry single variant analysis. **Figure S3.** Manhattan plot for EA single variant analysis. **Figure S4.** QQ plot for EA single variant analysis. **Figure S5.** Manhattan plot for AA single variant analysis. **Figure S6.** Quantile-quantile plot for AA single variant analysis. **Figure S7.** Miami plot European and African-American ancestry sex-stratified single variant analysis. **Figure S8.** Quantile-quantile plots for European and African-American ancestry sex-stratified single variant analyses. **Figure S9.** Normal morphology of adult *Adamts6* heterozygous hearts. (DOCX 4290 kb)

Additional file 3: **Video S1.** (Quicktime) Video to illustrate the DORV phenotype finding in an Adamts6 mutant heart. (MOV 1983 kb)
