# Supplementary material for: Exome-chip meta-analysis identifies novel loci associated with cardiac conduction, including ADAMTS6
Source: Genome Biol. 2018 Jul 17;19:87. doi: 10.1186/s13059-018-1457-6 (PMC6048820; doi:10.1186/s13059-018-1457-6)
Supplement: Supplementary file 2 — Figure S1. Manhattan plot for European and African-American ancestry single variant analysis. Figure S2. Quantile-quantile plot for European and African-American ancestry single variant analysis. Figure S3. Manhattan plot for EA single variant analysis. Figure S4. QQ plot for EA single variant analysis. Figure S5. Manhattan plot for AA single variant analysis. Figure S6. Quantile-quantile plot for AA single variant analysis. Figure S7. Miami plot European and African-American ancestry sex-stratified single variant analysis. Figure S8. Quantile-quantile plots for European and African-American ancestry sex-stratified single variant analyses. Figure S9. Normal morphology of adult Adamts6 heterozygous hearts. (DOCX 4290 kb) [file 13059_2018_1457_MOESM2_ESM.docx]

Exome-chip meta-analysis identifies novel loci associated with cardiac conduction, including ADAMTS6

# Supplementary Figures

1. Figure S1: Manhattan plot for European and African-American ancestry single variant analysis.
2. Figure S2: Quantile-quantile plot for European and African-American ancestry single variant analysis.
3. Figure S3: Manhattan plot for EA single variant analysis.
4. Figure S4: QQ plot for EA single variant analysis.
5. Figure S5: Manhattan plot for AA single variant analysis.
6. Figure S6: Quantile-quantile plot for AA single variant analysis.
7. Figure S7: Miami plot European and African-American ancestry sex-stratified single variant analysis.
8. Figure S8: Quantile-quantile plots for European and African-American ancestry sex-stratified single variant analyses.
9. Figure S9: Normal morphology of adult *Adamts6* heterozygous hearts.

**
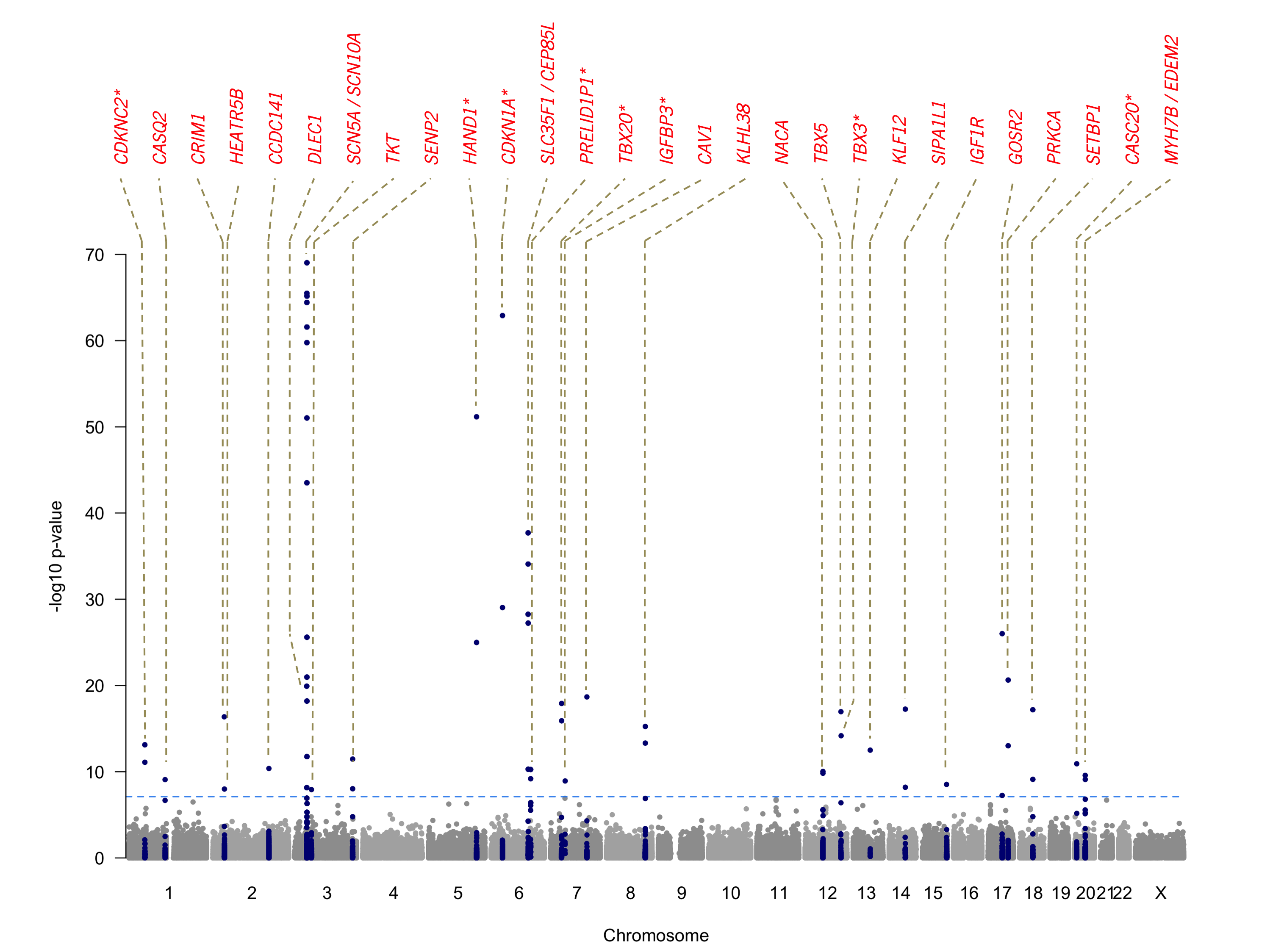
**

**Figure S1.** Manhattan plot for the combined European and African-American ancestry single variant meta-analysis. The horizontal axis represents chromosomal position and the vertical represents the association signal significance (–log10). Data points represents the relative chromosomal position and the –log10 p-value of variants included the analyses, where SNPs in blue are exome-wide significant (P<6.17x10^-8^), whereas those in grey did not pass this significance level, also represented by the blue dashed line. The lead SNPs in each independent locus are annotated with the names of the genes in which they reside, or the nearest gene in case these were intergenic, indicated in that case by an asterisk.

**
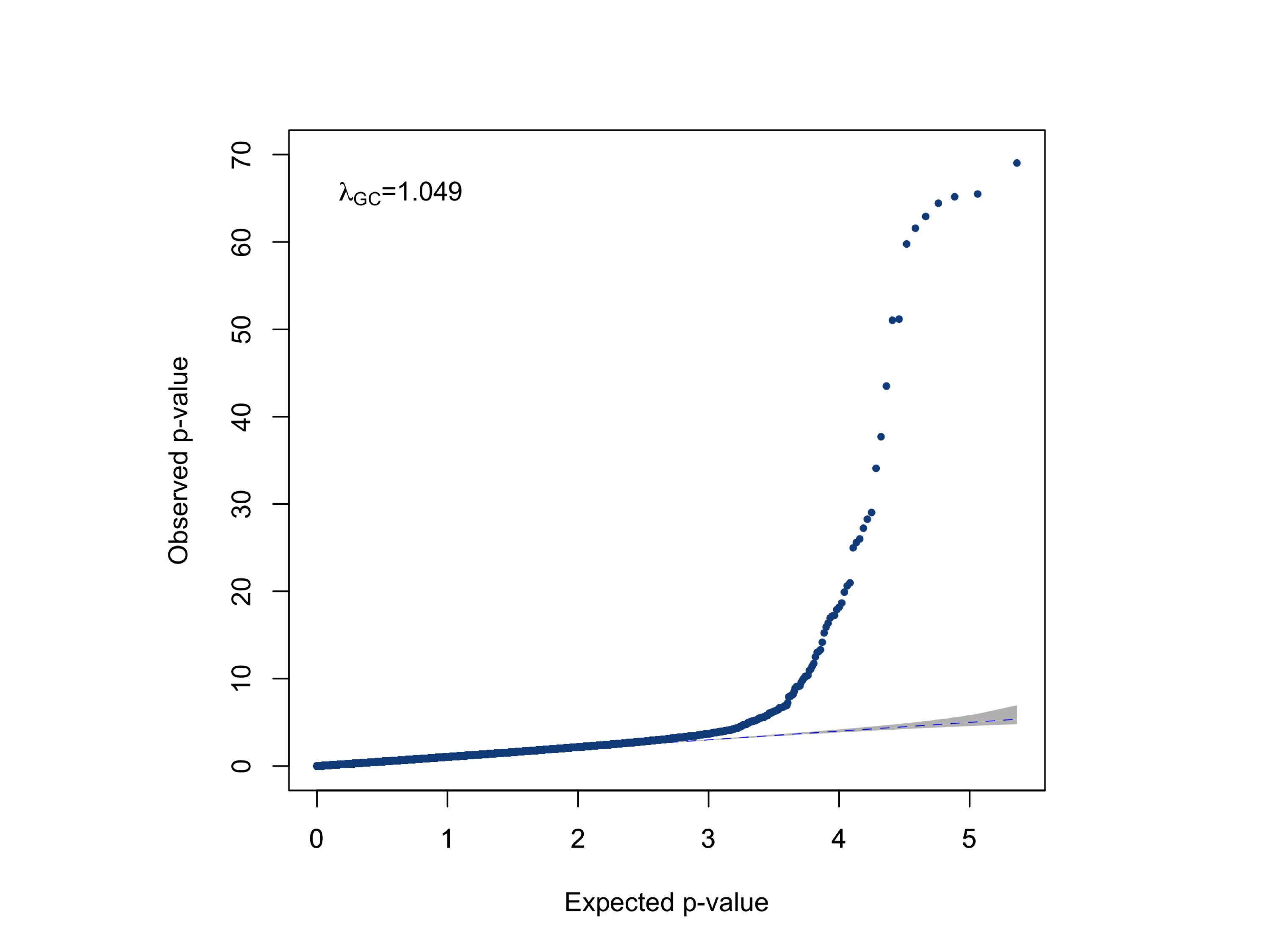
**

**Figure S2.** Quantile-quantile plot for p-values obtained from the combined European and African-American ancestry single variant meta-analysis. The horizontal and vertical axes represent the expected distribution of -log10(P-values) under the null hypothesis of no association, whereas the vertical axis shows the observed -log10(P-values). The blue dashed line represents the null, and λ_gc_ value represents the genomic inflation factor lambda. Each data point represents the observed versus the expected p-value of a variant included in the association analyses.

**
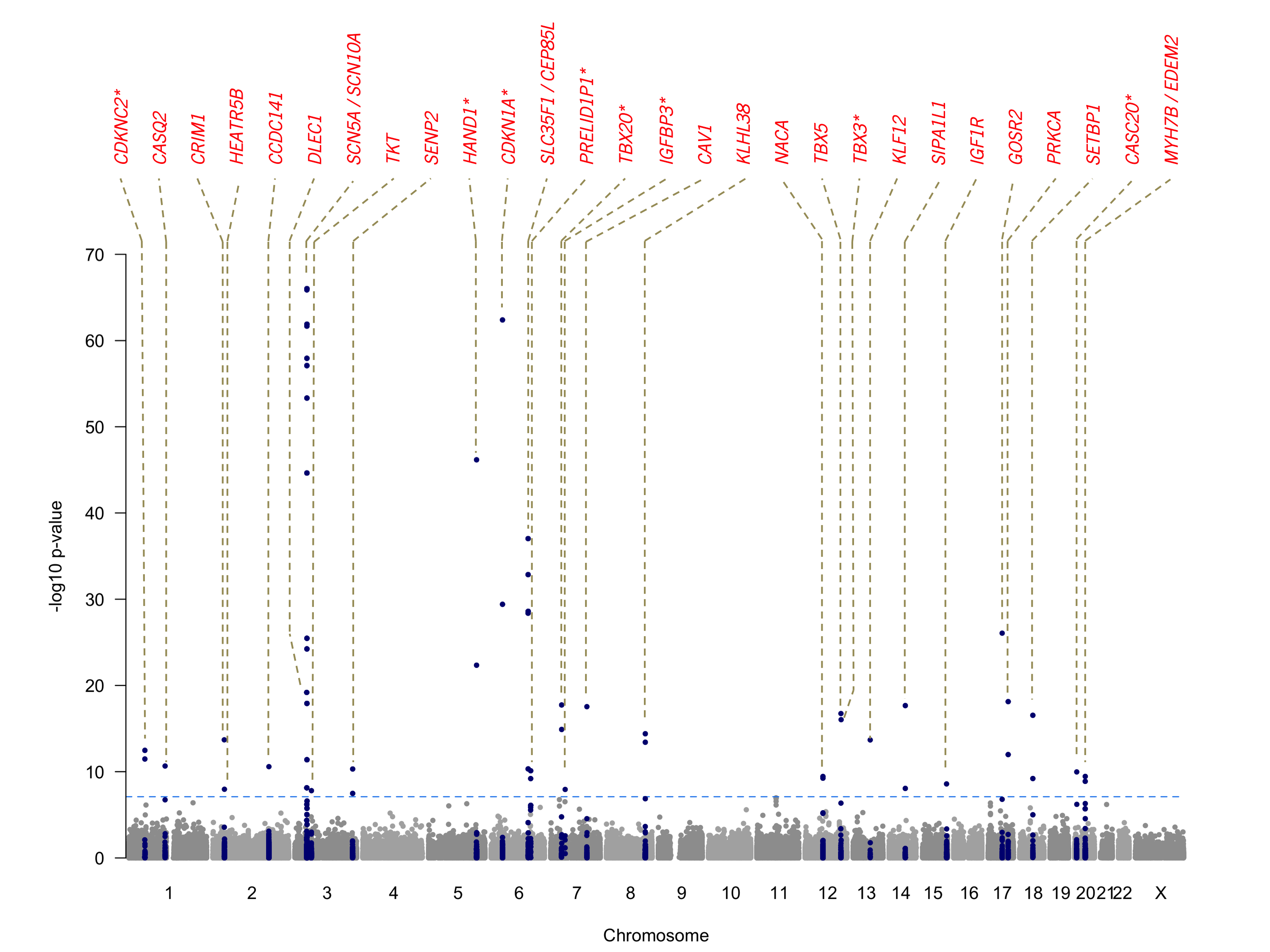
Figure S3.** Manhattan plot for the European ancestry single variant meta-analysis. The horizontal axis represents chromosomal position and the vertical represents the association signal significance (–log10). Data points represents the relative chromosomal position and the –log10 p-value of variants included the analyses, where SNPs in blue are exome-wide significant (P<6.10x10^-8^), whereas those in grey did not pass this significance level, also represented by the blue dashed line. The lead SNPs in each independent locus is annotated with the names of the genes in which they reside, or the nearest gene in case these were intergenic, indicated in that case by an asterisk.


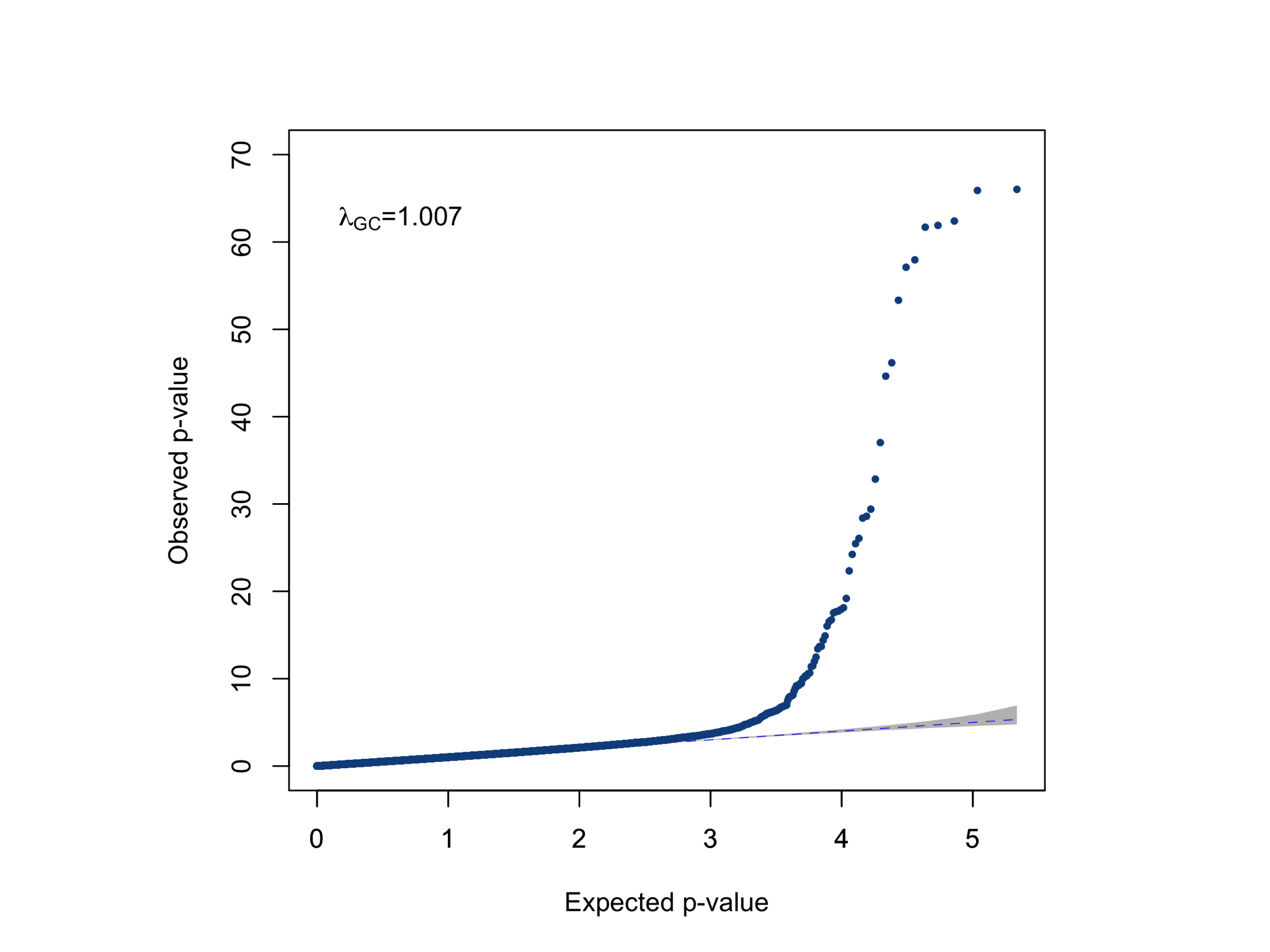


**Figure S4.** Quantile-quantile plot for p-values obtained from the European ancestry single variant meta-analysis. The horizontal and vertical axes represents the expected distribution of -log10(P-values) under the null hypothesis of no association, whereas the vertical axis shows the observed -log10(P-values). The blue dashed line represents the null, and λ_gc_ value represents the genomic inflation factor lambda. Each data point represents the observed versus the expected p-value of a variant included in the association analyses.

**
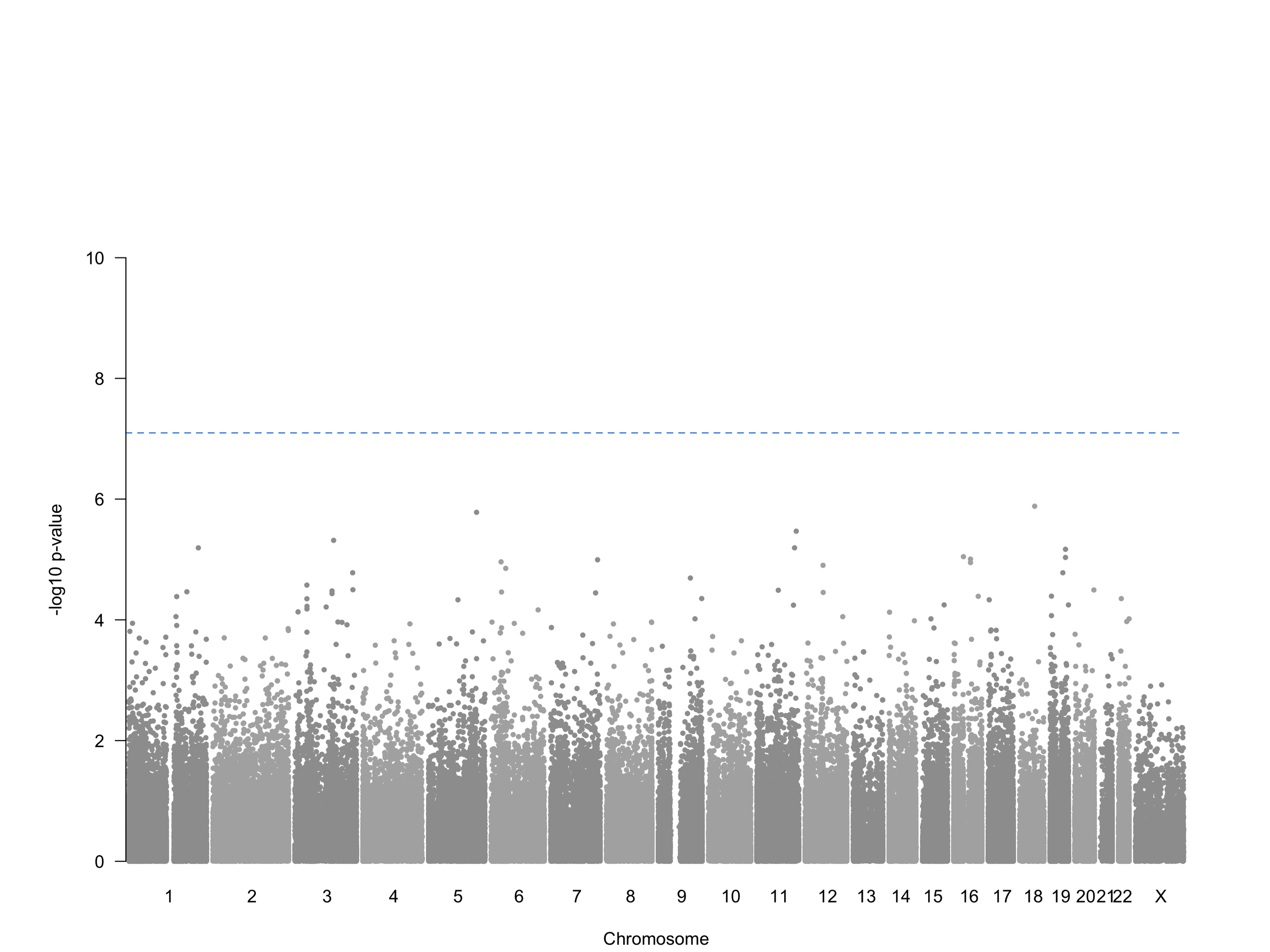
Figure S5.** Manhattan plot for the African-American ancestry single variant meta-analysis. The horizontal axis represents chromosomal position and the vertical axis represents the association signal significance (–log10). Data points represents the relative chromosomal position and the –log10 p-value of variants included the analyses, where SNPs in blue (non-existent here), are exome-wide significant (P<6.10x10^-8^), whereas those in grey did not pass this significance level, also represented by the blue dashed line.


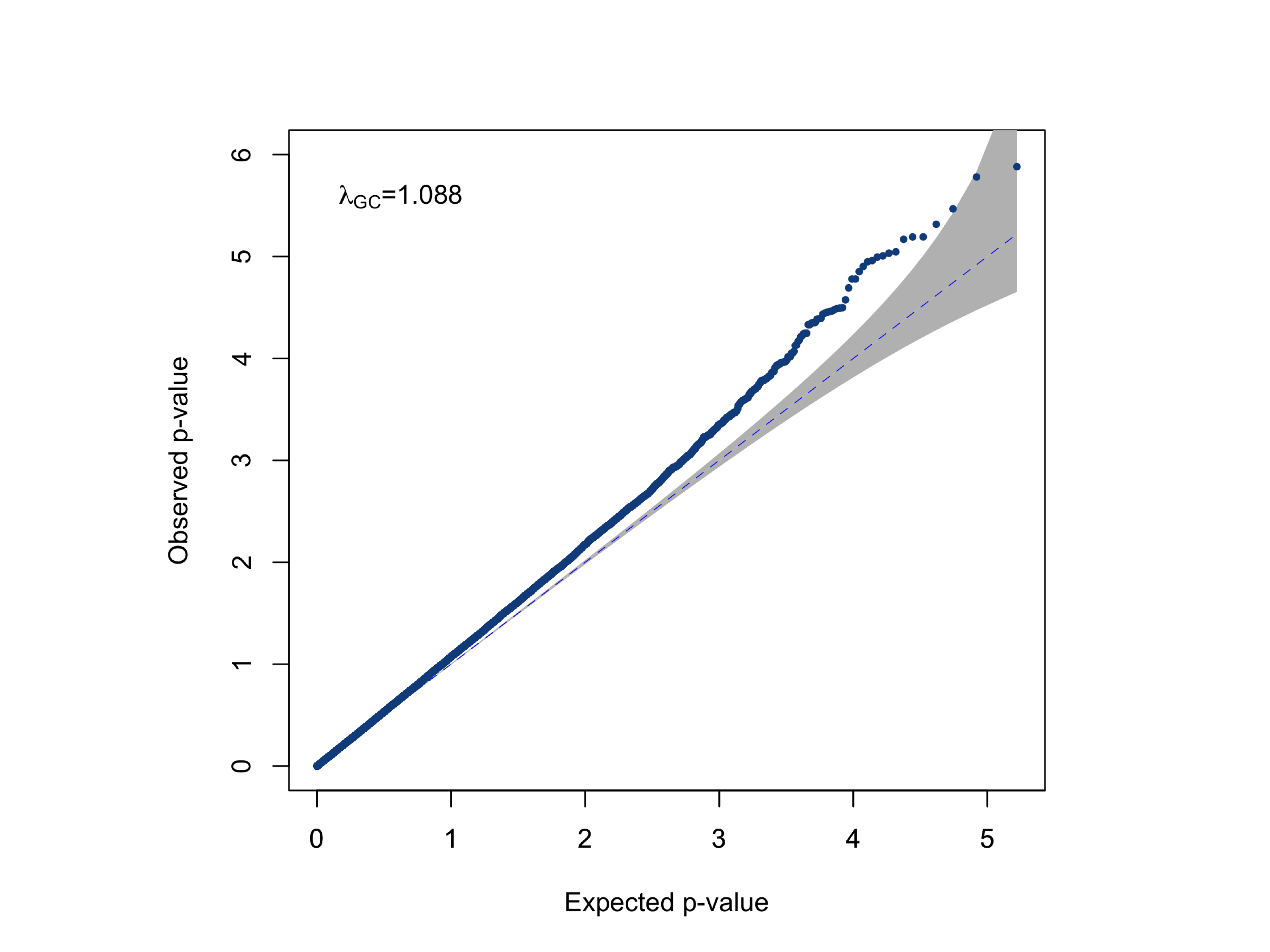


**Figure S6.** Quantile-quantile plot for p-values obtained from the African-American ancestry single variant meta-analysis. The horizontal and vertical axes represents the expected distribution of -log10(P-values) under the null hypothesis of no association, whereas the vertical axis shows the observed -log10(P-values). The blue dashed line represents the null, and λ_gc_ value represents the genomic inflation factor lambda. Each data point represents the observed versus the expected p-value of a variant included in the association analyses.

**
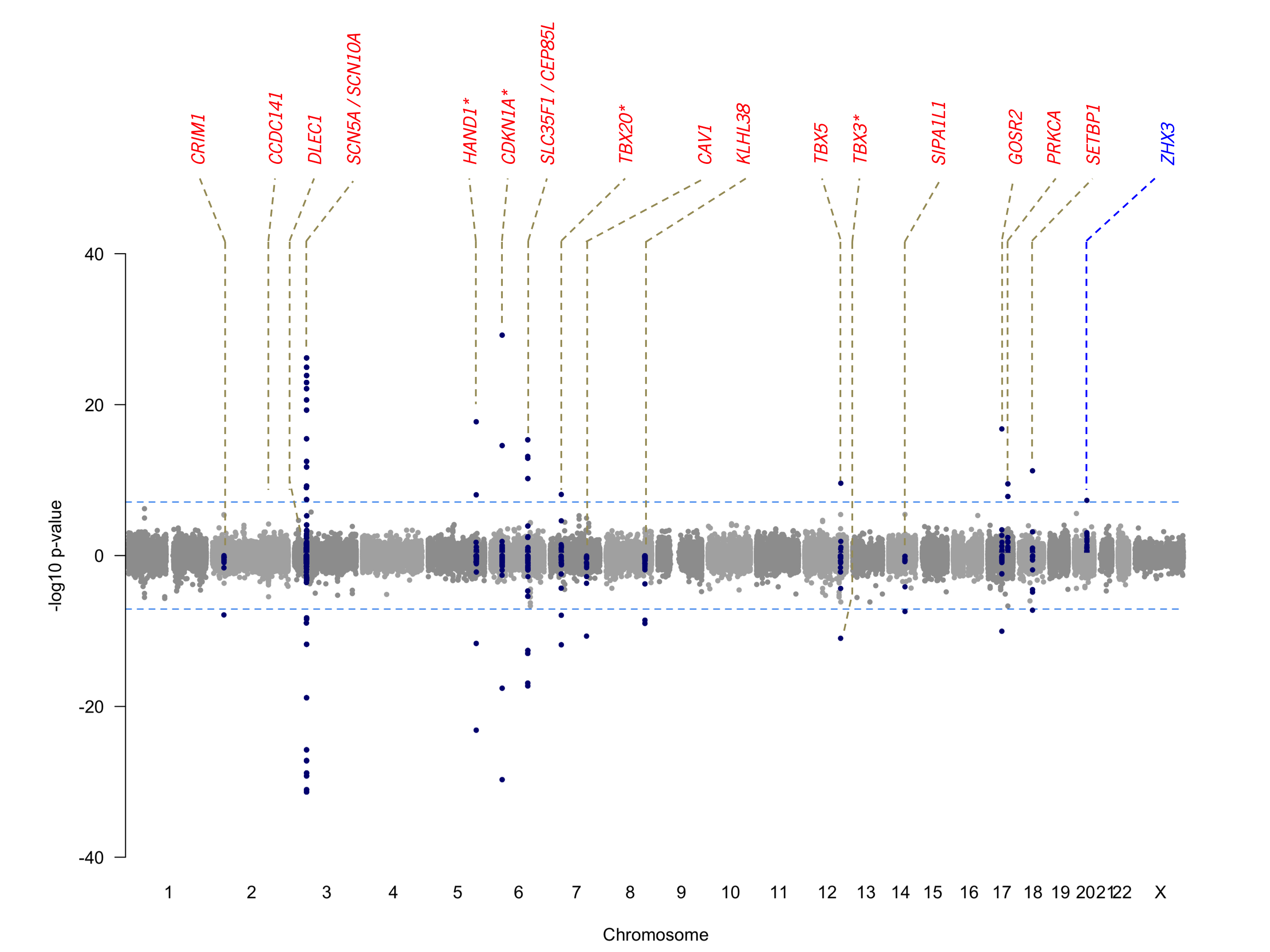
Figure S7.** Miami plot for the combined European and African-American ancestry single variant meta-analyses, stratified by sex. The horizontal axis represents chromosomal position whereas the lower vertical axis represents the association signal significance (–log10) for the association analysis for males only, and the upper vertical axis represents the association signal significance (–log10) for the association analysis for females only. Data points represents the relative chromosomal position and the –log10 p-value of variants included the analyses, where SNPs in blue are exome-wide significant (P<6.10x10^-8^), whereas those in grey did not pass this significance level, also represented by the light blue horizontal dashed line. The lead SNPs in each independent locus is annotated with the names of the genes in which they reside, or the nearest gene in case these were intergenic, indicated in that case by an asterisk. The one variant that showed a sex-specific effect size different (rs17265513 (C/T), in *ZHX3*, on chromosome 20), is annotated using a blue dashed vertical line.


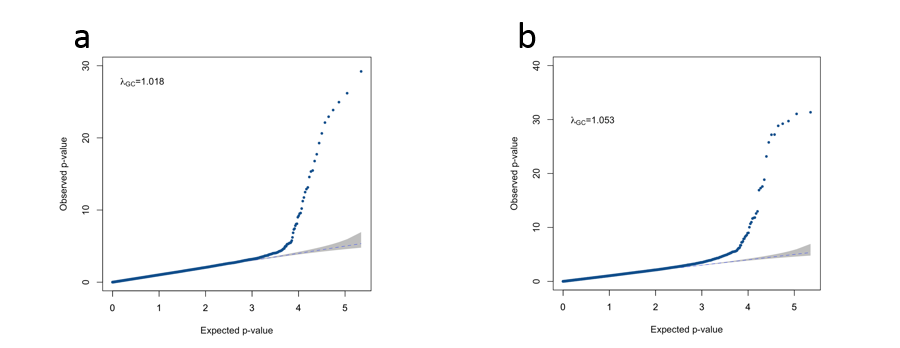


**Figure S8.** Quantile-quantile plots for European and African-American ancestry sex-stratified single variant analyses. (**a**) QQ plot for the male-only analysis and (**b**) the QQ plot for the female-only analysis. The horizontal and vertical axes represents the expected distribution of -log10(P-values) under the null hypothesis of no association, whereas the vertical axis shows the observed -log10(P-values). The blue dashed line represents the null, and λ_gc_ value represents the genomic inflation factor lambda. Each data point represents the observed versus the expected p-value of a variant included in the association analyses.

**
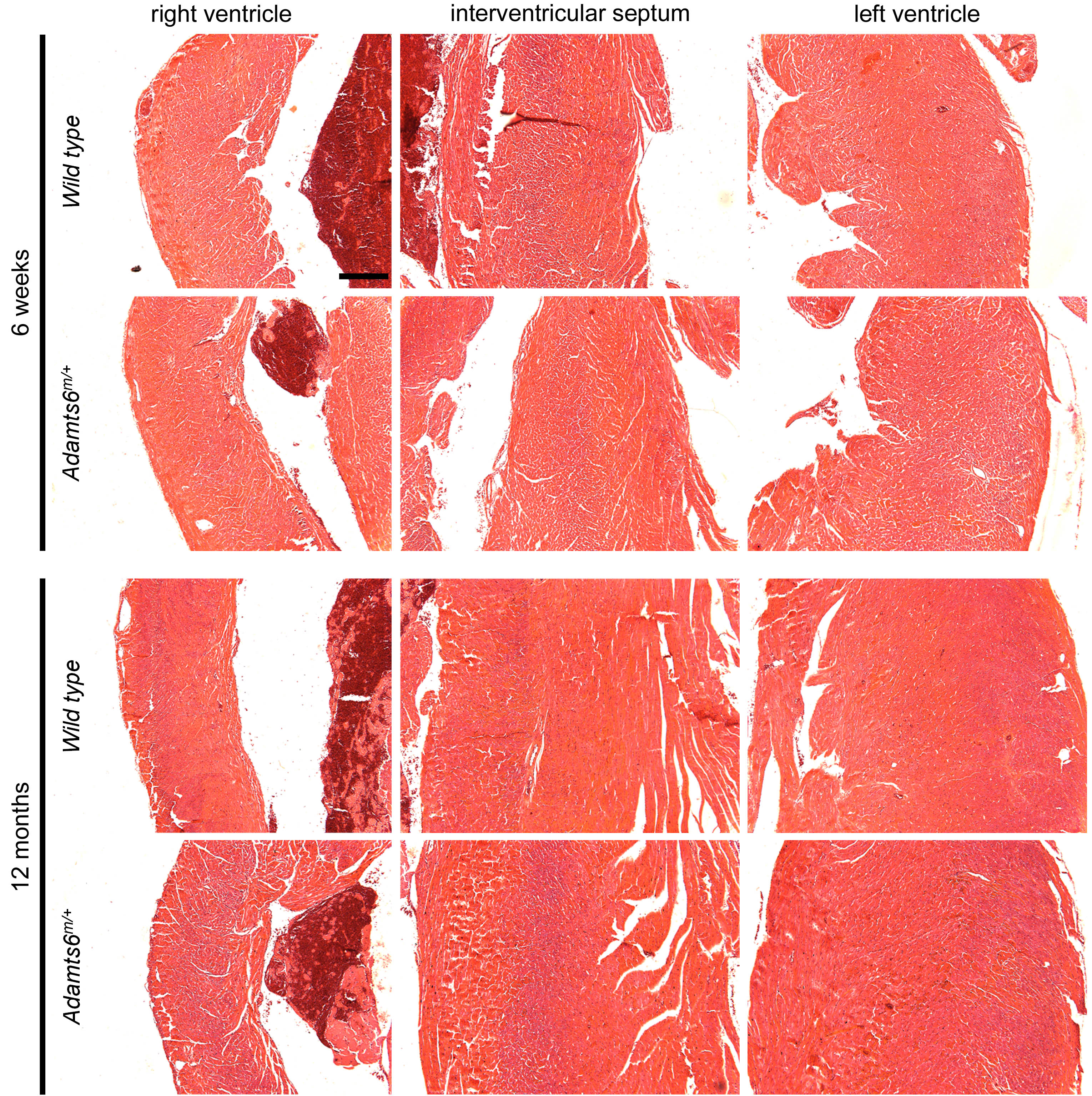
**

**Figure S9.** Ventricular myocardial morphology is unchanged in *Adamts6*-deficient mice. Hematoxylin and eosin stained hearts show comparable myocardium size and structure in 6 week and 12 month-old *Adamts6^m/+^* mice as compared to wild type. Scale bar: 50μm.
